# Mechanostimulatory Cues Determine Intestinal Fibroblast Fate and Profibrotic Remodeling in a Physiodynamic Human Gut‐on‐a‐Chip

**DOI:** 10.1002/advs.202516040

**Published:** 2026-04-10

**Authors:** Soyoun Min, Nam Than, Yong Cheol Shin, Elif G. Ertugral, Chandrasekhar R. Kothapalli, Olumuyiwa Awoniyi, Hyun Jung Kim

**Affiliations:** ^1^ Department of Inflammation and Immunity Cleveland Clinic Research Cleveland Clinic Cleveland Ohio USA; ^2^ Department of Chemical and Biomedical Engineering Cleveland State University Cleveland Ohio USA; ^3^ Digestive Disease Institute Department of Gastroenterology Hepatology, and Nutrition Cleveland Clinic Cleveland Ohio USA; ^4^ Cleveland Clinic Lerner College of Medicine of Case Western Reserve University School of Medicine Cleveland Ohio USA

**Keywords:** epithelial barrier dysfunction, fibroblast activation, fluid shear stress, gut‐on‐a‐chip, inflammatory bowel disease, intestinal fibrosis, mechanoadaptation

## Abstract

Biomechanical cues such as fluid shear stress and mechanical strain regulate intestinal physiology, yet their roles in shaping fibroblast fate during early fibrotic remodeling remain poorly defined. Here, we use a microengineered human gut‐on‐a‐chip model that enables independent control of shear stress and mechanical strain under conditions of intact or impaired epithelial barrier function to interrogate fibroblast dynamics. Inflammation‐associated fibroblasts derived from an ulcerative colitis patient exhibit intrinsic tolerance to biomechanical stress, maintaining myofibroblast‐like phenotypes marked by hypertrophy and elevated α‐smooth muscle actin aligned with actin stress fibers. In contrast, normal fibroblasts from healthy donors are highly susceptible to fluid shear stress, undergoing matrix metalloproteinase‐dependent disruption of focal adhesion signaling, extracellular matrix remodeling, and apoptotic cell death, whereas mechanical strain alone exerts minimal effects. Importantly, an intact epithelial barrier is necessary and sufficient to protect fibroblasts from shear‐induced injury, suggesting that “good fences make good neighbors.” Under barrier dysfunction, prolonged shear exposure promotes the emergence of stiff 3D aggregates composed of mechanoadaptive, myofibroblast‐like cells embedded within a complex fibrillar network. These findings identify that fluid shear stress contributes as a key driver of early profibrotic remodeling and highlight epithelial barrier integrity as a critical biomechanical safeguard in inflammatory bowel disease.

## Graphical abstract

A healthy gut barrier shields underlying fibroblasts from luminal shear forces, illustrating that “good fences make good neighbors.” Barrier damage exposes fibroblasts to shear stress, inducing cell death and the emergence of stress‐adapted, profibrotic fibroblasts. Sustained shear exposure promotes the formation of stiff aggregates of mechanoadapative fibroblasts, driving tissue scarring and early fibrotic remodeling in inflammatory bowel disease. White bar, 50 µm; Yellow bar, 10 µm.

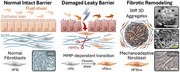

## 1 Introduction

Intestinal fibrosis in inflammatory bowel disease (IBD) results from recurrent and localized inflammatory injury, leading to tissue stiffening, scarring, and structural complications such as strictures, fistulas, and luminal narrowing [1, 2]. In both Crohn's disease (CD) and ulcerative colitis (UC), mucosal fibrosis is linked to chronic inflammation and epithelial barrier dysfunction, exposing underlying fibroblasts to abnormal luminal fluid shear and disrupting extracellular matrix (ECM) remodeling, ultimately impairing bowel function [3]. Despite its high prevalence, no targeted therapies exist for IBD‐associated fibrosis, as its primary drivers remain unclear [4]. Notably, a subset of IBD patients, prevalently in CD, develops severe fibrosis and exhibits poor responsiveness to biologic therapies, including anti‐tumor necrosis factor (TNF) blockers [5, 6, 7, 8], indicating that fibrosis progression is not solely driven by inflammation. Hence, identifying key profibrotic triggers beyond inflammation is essential to advance our understanding of early fibrogenic processes and develop effective anti‐fibrotic therapies.

The intestinal mucosa is inherently subjected to rhythmic mechanical deformations and luminal shear stress during peristalsis, which are essential for maintaining intestinal homeostasis [9]. In IBD, chronic inflammation often disrupts the epithelial barrier, creating damaged mucosal loci that serve as initiation sites for early fibrotic remodeling [10]. Epithelial barrier dysfunction exacerbates biomechanical stress on subepithelial fibroblasts, exposing them to abnormal physical forces. However, the extent to which these forces drive fibroblast activation, excessive ECM deposition [11], secretion of fibrogenic cytokines [12], responsiveness to microbial signals [13], and differentiation into myofibroblasts [14] remains poorly understood. Furthermore, dysregulated intestinal peristalsis in IBD further amplifies mechanobiological stress on stromal cells, often associated with abnormalities in intestinal smooth muscle cells (SMCs) [15, 16, 17]. Despite these associations, the specific contribution of luminal shear stress and mechanical deformation to early fibrotic cascades remains unclear. Notably, investigating the independent role of pathobiological and biomechanical factors has been hindered by the confounding complexity of in vivo systems. Hence, a modular intestine model capable of recapitulating the mucosal microenvironment and mechanobiological interactions is essential to elucidate the mechanostimulatory cues governing fibroblast activation and profibrotic remodeling in IBD.

We have developed a bioengineered human gut‐on‐a‐chip microphysiological system (MPS) that can demonstrate 3D morphogenesis of intestinal epithelial cells [18, 19, 20, 21], peristalsis‐like physiodynamic motions and fluid shear stress [22, 23, 24], multi‐cellular host‐microbiome co‐cultures including anaerobic gut bacteria [22, 25], pathomimetic modeling of human intestinal disorders [26, 27, 28], and integrative cultures with human intestinal organoids for conducting precision medicine studies [23, 24]. This modular system facilitates the mechanistic identification of specific pathophysiological factors by selectively introducing or removing target cell types or contributing components in a controlled spatiotemporal manner. For example, repetitive deformations in a gut‐on‐a‐chip are designed to emulate the mechanical contribution of SMCs in the human intestine. This approach aims to isolate the mechanobiological effects of SMCs on intestinal mucosal cells, eliminating confounding biological factors and reducing in vivo complexity for mechanistic studies. Additionally, the gut‐on‐a‐chip enables independent control of fluid flow and cyclic stretching, providing a detailed assessment of the mechanomodulatory milieu affecting mucosal cells in a pathological context. Notably, replicating these biomechanical cues in animal models or conventional cell culture models remains a significant challenge.

In this study, we employed a physiodynamic gut‐on‐a‐chip model to interrogate how biomechanical cues contribute to the early stages of intestinal fibrosis. By independently modulating fluid shear stress and mechanical deformation, we assessed how these mechanostimulatory factors influence fibroblast reprogramming and drive profibrotic remodeling. We further investigated the role of an intact epithelial barrier in regulating mechanodynamic stress during the initiation of the profibrotic process in IBD. Our findings suggest that this modular gut‐on‐a‐chip model may offer a powerful and physiologically relevant framework for recapitulating profibrotic microenvironments, dissecting key mechanobiological drivers of fibrosis, and enabling the identification of potential therapeutic targets.

## 2 Results

### 2.1 Inflammation‐Associated Fibroblasts Retain Disease‐Specific Cellular Characteristics

**FIGURE 1:**
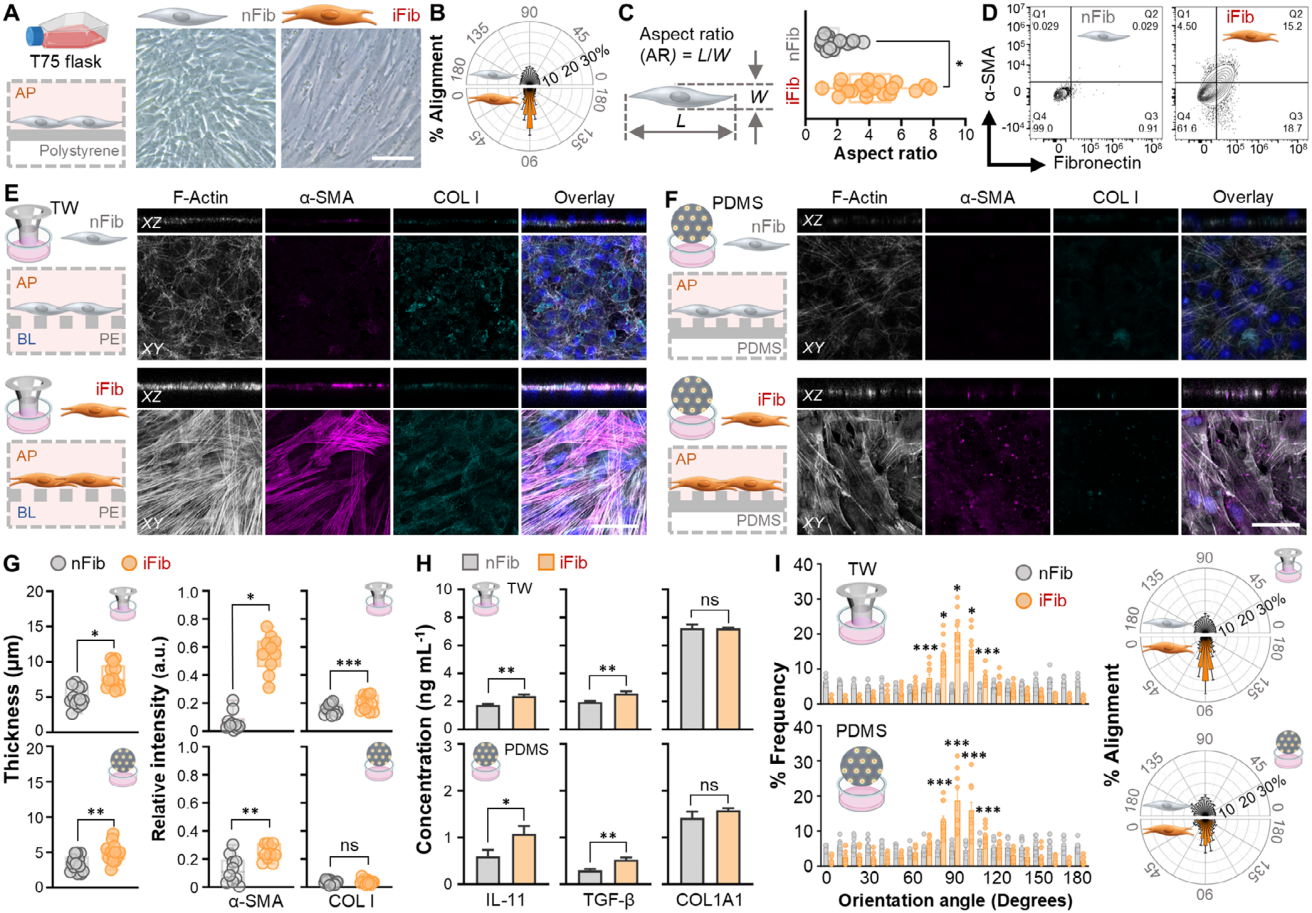
Inflammation‐associated fibroblasts retain profibrotic characteristics influenced by substrate stiffness and culture conditions. (A) Phase‐contrast micrographs of normal fibroblasts (nFib) and inflammation‐associated fibroblasts (iFib) cultured on rigid T75 flask surfaces for 110 h, selected as the endpoint for baseline phenotypic characterization based on prior optimization. (B) Orientation index of nFib and iFib quantified by probability‐based alignment analysis on T75 flasks after 110 h (*n* = 3). (C) Aspect ratio distributions of nFib and iFib cultured on T75 flasks for 110 h (*n* = 20). (D) Flow cytometric analysis of nFib and iFib cultured on T75 flasks, highlighting fibrotic markers α‐SMA and fibronectin. (E) Morphological and functional characteristics of nFib and iFib cultured on a Transwell (TW) with polyester nanoporous inserts (pore size: 0.4 µm) for 5 days. Immunofluorescence micrographs show top‐down (*XY*) and cross‐sectional (*XZ*) views with markers for F‐actin (gray), α‐SMA (magenta), COL I (cyan), and their overlay with nuclei (blue) (*n* = 5). (F) Morphological and functional features of nFib and iFib cultured on PDMS membranes (10:1 w/w, prepolymer: curing agent) mounted on glass‐bottom plates for 5 days, visualized using the same immunofluorescence markers as in panel E. (G) Quantification of cell‐layer thickness (left) and relative fluorescence intensity of indicated markers (right) from panels E and F (*n* = 5). (H) Production of fibrosis‐associated proteins (IL‐11, TGF‐β, and COL1A1) by nFib and iFib cultured on Transwells or PDMS membranes for 5 days, measured by ELISA (*n* = 5). (I) Quantitative analysis of cellular orientation in nFib and iFib cultured on Transwells or PDMS membranes for 5 days. Left, frequency distribution of orientation angles; right, percentage alignment profiles (*n* = 5). Data in box plots (1C and 1G) show median with min‐to‐max whiskers. Data in column charts (1B, 1H, and 1I) are presented as mean ± SEM. Statistical significance was determined using an unpaired, two‐tailed Student's *t*‐test (1B, 1C, 1G, 1H, and 1I). Bars, 50 µm. ^*^ *p*<0.001, ^**^ *p*<0.01, ^***^ *p*<0.05. ns, not significant.

To investigate the mechanostimulatory responses of intestinal fibroblasts in the context of profibrotic processes in IBD, we analyzed the morphological and functional characteristics of normal fibroblasts (nFib) and inflammation‐associated fibroblasts (iFib), isolated from a healthy donor and a colonic biopsy of a UC patient with severe inflammatory injury, respectively. When these cells were cultured on a rigid surface of a standard T75 flask (stiffness at 3.07±0.18 GPa, Figure S1A), nFib exhibited random orientations and typical fibroblast morphology, whereas iFib displayed a striped, elongated appearance (Figure 1A; Figure S1B). At confluence, nFib maintained a stochastic orientation, while iFib demonstrated anisotropic alignment with a defined orientation index (Figure 1B; Figure S1B). Quantitative analysis of cell shapes revealed that iFib had a significantly higher aspect ratio (4.20±0.41, *p*<0.001) compared to nFib (1.78±0.17), confirming distinct morphological differences between the two fibroblast types (Figure 1C). Furthermore, molecular profiling of nFib and iFib cultured on T75 flasks confirmed that iFib exhibited elevated expression of α‐smooth muscle actin (α‐SMA) and fibronectin among CD90^+^ and vimentin^+^ cells, as quantified by flow cytometry (Figure 1D; Figure S1C). These findings confirmed the distinct morphological and molecular characteristics of inflammation‐associated fibroblasts compared to normal fibroblasts.

The stiffness of the culture substrate significantly influences the morphological and functional characteristics of intestinal fibroblasts [29]. We evaluated the behavior of nFib and iFib cultured on substrates softer than conventional tissue culture flasks. When nFib were cultured for 5 days on a polyester nanoporous membrane in a Transwell (stiffness: 174.34±16.47 MPa; Figure S1A), they exhibited disorganized and weakly developed F‐actin fibers, low collagen I (COL I) expression, and an absence of detectable α‐SMA expression (Figure 1E, upper). Addition of profibrotic molecules, such as interleukin 11 (IL‐11; 10 ng mL^−1^) [30] and transforming growth factor beta (TGF‐β; 10 ng mL^−1^) [31], to nFib cells cultured in Transwells resulted in substantial upregulation of COL I, but no detectable α‐SMA expression (Figure S1E). In contrast, iFib displayed significantly elevated α‐SMA expression co‐localized with organized actin stress fibers (*p*<0.001) (Figure 1E, lower and Figure S1D). On softer polydimethylsiloxane (PDMS) silicone membranes (stiffness: 20.67±1.13 kPa; Figure S1A), nFib cells showed marked reductions in F‐actin expression (Figure 1F, upper) and cell layer thickness (Figure 1G, left), along with significantly decreased α‐SMA (∼8.18‐fold, *p*<0.001) and COL I expression (∼1.30‐fold, *p*<0.01) compared to those on Transwell membranes (Figure 1E,F). Notably, treatment with a profibrotic cocktail of IL‐11 and TGF‐β did not induce detectable α‐SMA, although COL I expression was substantially increased (Figure S1E). Cross‐sectional analysis revealed that iFib formed significantly thicker cell layers than nFib on both Transwell (∼1.50‐fold; *p*<0.001) and PDMS substrates (∼1.46‐fold; *p*<0.01) (Figure 1G, left). Additionally, iFib secreted significantly higher levels of fibrosis‐associated cytokines, including IL‐11 and TGF‐β, across both culture substrates, whereas the secretion of collagen type I alpha 1 (COL1A1) did not significantly differ between nFib and iFib (Figure 1H). Morphologically, nFib exhibited random, non‐aligned growth, whereas iFib consistently displayed pronounced directional alignment, with orientation angles skewed toward 90° regardless of substrate stiffness (Figure 1I). These findings confirm the distinct biochemical and morphological characteristics of nFib and iFib, demonstrating that iFib robustly maintains disease‐intrinsic features in vitro.

### 2.2 Biomechanical Cues Amplify Profibrotic Signatures in Inflammation‐Associated Fibroblasts

**FIGURE 2:**
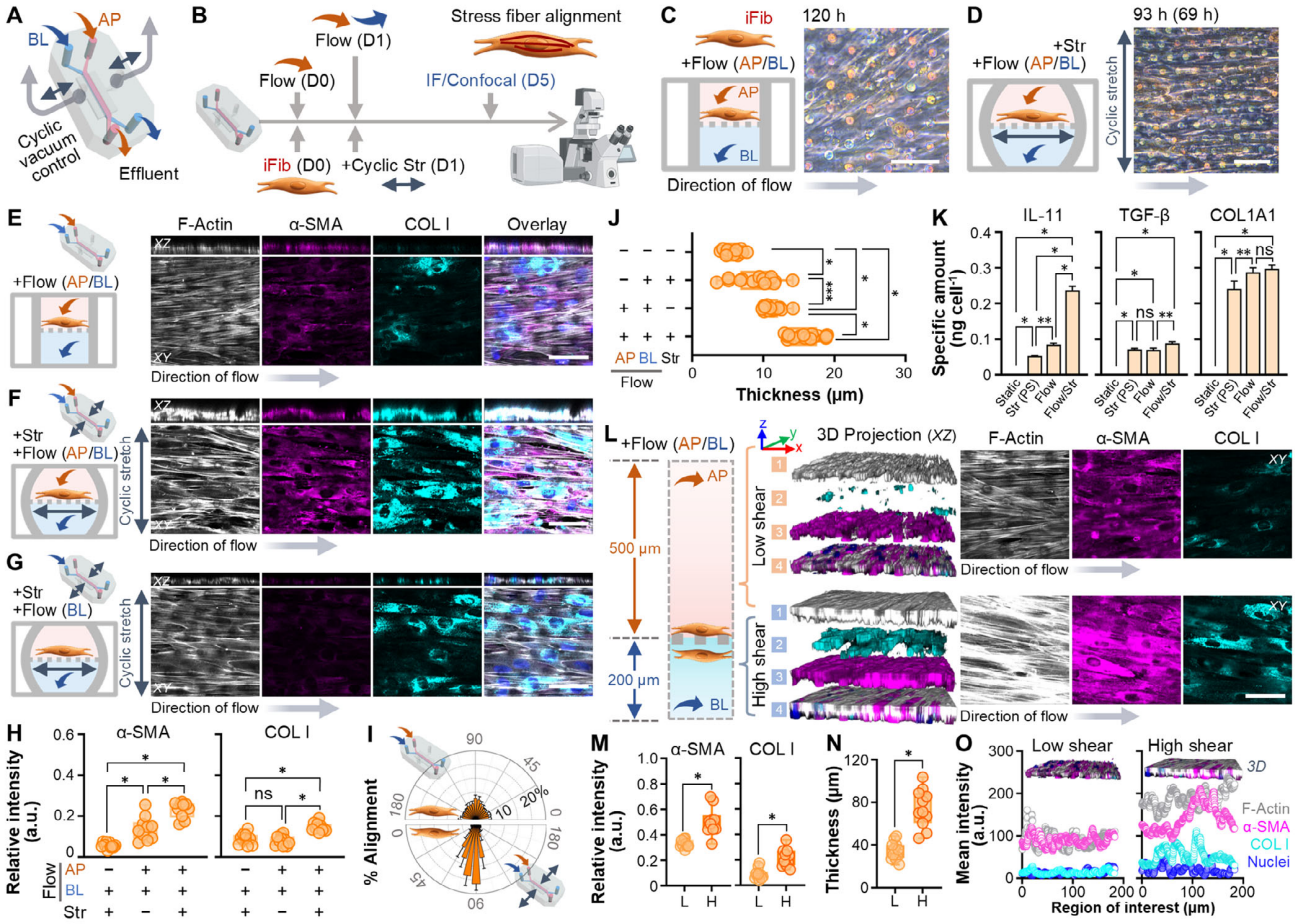
Fluid shear stress and cyclic mechanical strain promote profibrotic responses in inflammation‐associated fibroblasts in a gut‐on‐a‐chip. (A) Schematic of the gut‐on‐a‐chip setup, illustrating independent manipulation of biomechanical cues, including fluid shear stress applied to the apical (AP; orange arrow) and basolateral (BL; blue arrow) microchannels and cyclic mechanical strain (double arrows). Gray arrows indicate the repetitive pneumatic actuation used to generate cyclic deformation. (B) Experimental workflow depicting exposure of iFib to defined biomechanical cues followed by immunofluorescence (IF) microscopic analysis of profibrotic phenotypes, including actin stress fibers alignment with α‐SMA. Experimental time points are indicated as Days 0, 1, and 5 (D0, D1, and D5). (C) Phase‐contrast micrograph showing robust iFib growth under fluid shear stress (20 µL h^−1^ for 120 h) applied simultaneously to both AP and BL microchannels. (D) Phase‐contrast image illustrating perpendicular alignment of iFib subjected to cyclic mechanical strain (+Str) for 69 h, selected to capture time‐dependent cellular responses prior to the maximum feasible culture duration under microfluidic dual flow (20 µL h^−1^; +Flow AP/BL; total culture time of 93 h). (E) Confocal IF images of iFib exposed to microfluidic flow alone applied to both AP and BL microchannels (20 µL h^−1^; total culture time of 125 h), showing increased expression of profibrotic markers α‐SMA (magenta) and COL I (cyan) relative to static PDMS membrane controls shown in Figure 1F. (F) Combined microfluidic flow (20 µL h^−1^) and cyclic mechanical deformation (duration: 101 h) further enhanced α‐SMA and COL I expression, co‐localization with actin stress fibers (F‐actin; gray), cellular elongation, and increased cell height. (G) Application of microfluidic flow to the BL channel alone (20 µL h^−1^; total culture time of 125 h) under cyclic stretching induces perpendicular alignment of iFib relative to the stretch axis. (H) Quantification of α‐SMA and COL I fluorescence intensity under the conditions shown in panels E and F (*n* = 10). (I) Orientation index analysis demonstrating increased alignment probability of iFib under cyclic deformation (*n* = 10). (J) Quantification of cell‐layer thickness from IF images in panels E‐G; static control data are adapted from Figure 1F (*n* = 10). (K) Production of fibrosis‐associated proteins (IL‐11, TGF‐β, and COL1A1) by iFib cultured in the gut‐on‐a‐chip under pseudostatic stretch (Str PS), Flow, or combined Flow/Str, as well as static PDMS membrane control (Static) for 5 days, measured by ELISA. (*n* = 3). (L) Effect of fluid shear stress on iFib cultured on both sides of a porous PDMS membrane in the gut‐on‐a‐chip, comparing low‐ (∼0.00133 dyne cm^−2^) and high‐shear (∼0.00833 dyne cm^−2^) regimes generated by a flow rate of 20 µL h^−1^ for 125 h. (M) Relative fluorescence intensity of α‐SMA and COL I in iFib challenged to low (L) and high (H) shear stress in a gut‐on‐a‐chip (*n* = 3). (N) Quantification of cell‐layer thickness under low (L) and high (H) shear conditions from panel L (*n* = 10). (O) Mean fluorescence intensity of iFib cultured under low (L) and high (H) shear stress as a function of imaging location within the gut‐on‐a‐chip. Overlaid 3D projection insets were adapted from panel L. Data in box plots (2J, 2H, 2 M, and 2N) show median with min‐to‐max whiskers. Data in column charts (2K and 2I) are presented as mean ± SEM. Statistical significance was determined using an unpaired, two‐tailed Student's *t*‐test (2I, 2 M, and 2N) or one‐way ANOVA with Turkey's multiple comparisons test (2H, 2J, and 2K). XY, top‐down views; XZ, vertical cross‐sectional views. Bars, 50 µm. ^*^ *p*<0.001, ^**^ *p*<0.01, ^***^ *p*<0.05. ns, not significant.

We next investigated how mechanobiological cues, such as fluid shear stress and peristalsis‐like cyclic mechanical strain, influence the cellular and molecular signatures of inflammation‐associated fibroblasts under conditions of epithelial barrier impairment. To recapitulate this pathophysiological microenvironment, we systematically decoupled and reconstituted these biomechanical stimuli into three distinct regimes: fluid shear stress alone (+Flow), cyclic mechanical strain alone (+Str), or a combination of both (+Flow, +Str), directly applied to iFib cells cultured in a modular gut‐on‐a‐chip (Figure 2A; Figure S2A and Movies S1 and S2). iFib cells were seeded in the upper microchannel of the gut‐on‐a‐chip and subjected to controlled microfluidic flow (e.g., apical (AP), basolateral (BL), or both; 20 µL h^−1^ volumetric flow rate) and cyclic stretch induced by repetitive pneumatic vacuum suction (10% cell strain, 0.15 Hz frequency; Figure S2B) throughout the culture period (Figure 2B).

Under fluidic flow applied to both the AP and BL microchannels without mechanical stretching (Fluidic), iFib cells exhibited spindle‐shaped morphologies with random orientations (Figure 2C). However, when both fluid shear stress and cyclic stretch were applied simultaneously (Dynamic), the cells aligned perpendicularly to the direction of repetitive stretch even after 69 h of exposure (Figure 2D). The “Fluidic” condition induced significantly elevated α‐SMA and COL I expression (Figure 2E,H) compared to static cultures (Figure 1F,G). Under “Dynamic” conditions, iFib displayed a pronounced hypertrophic response characterized by increased cell layer thickness (Figure 2F,J), upregulated α‐SMA and COL I expression (Figure 2F,H), and co‐localization of actin stress fibers with α‐SMA. Additionally, iFib cells demonstrated a strong orientational alignment in response to combined stimulation with fluid flow and cyclic mechanical strain (Figure 2I). Conversely, under pseudostatic BL flow conditions without apical fluid shear, repetitive stretching alone did not significantly increase α‐SMA expression (Figure 2G,H), and cell layer thickness was markedly reduced (Figure 2G,J), suggesting that apical fluid shear stress is a critical determinant for α‐SMA induction in iFib. Mechanical strain, however, consistently enhanced COL I deposition across all conditions (Figure 2F,H), supporting its role in promoting ECM production. The secretion of profibrotic molecules, such as IL‐11, TGF‐β, and COL1A1, was maximized when fluid shear stress and mechanical strain coexisted (Figure 2K). These results underscore the synergistic effects of biomechanical cues in amplifying profibrotic behaviors of iFib.

To assess the effect of fluid shear stress, iFib cells were seeded on a porous membrane in opposing orientations within a gut‐on‐a‐chip, where the upper and lower microchannels had distinct heights of 500 and 200 µm, respectively. This height differential generated surface shear stress at approximately 1.33 × 10^−3^ and 8.33 × 10^−3^ dyne cm^−2^ in the upper and lower microchannels, respectively, resulting in a 6.25‐fold shear stress gradient when a volumetric flow rate of 20 µL h^−1^ was applied to both channels. Under these conditions, iFib cells predominantly aligned in the direction of flow, exhibiting significantly increased α‐SMA (∼1.53‐fold; *p*<0.001) and COL I (∼2.46‐fold; *p*<0.001) expression in the high‐shear lower microchannel (Figure 2L,M). Moreover, cell layer thickness was markedly greater (∼2.13‐fold; *p*<0.001) in the high‐shear regime (Figure 2N), accompanied by enhanced actin stress fiber formation co‐localized with α‐SMA (Figure 2O). These findings confirmed that iFib cells are both tolerant and stable to biomechanical cues, exhibited shear‐dependent profibrotic phenotypes, where elevated biomechanical stimuli drive enhanced ECM production and cytoskeletal remodeling in vitro.

### 2.3 Apical Shear Stress Drives Atrophic Damage in Normal Intestinal Fibroblasts

**FIGURE 3:**
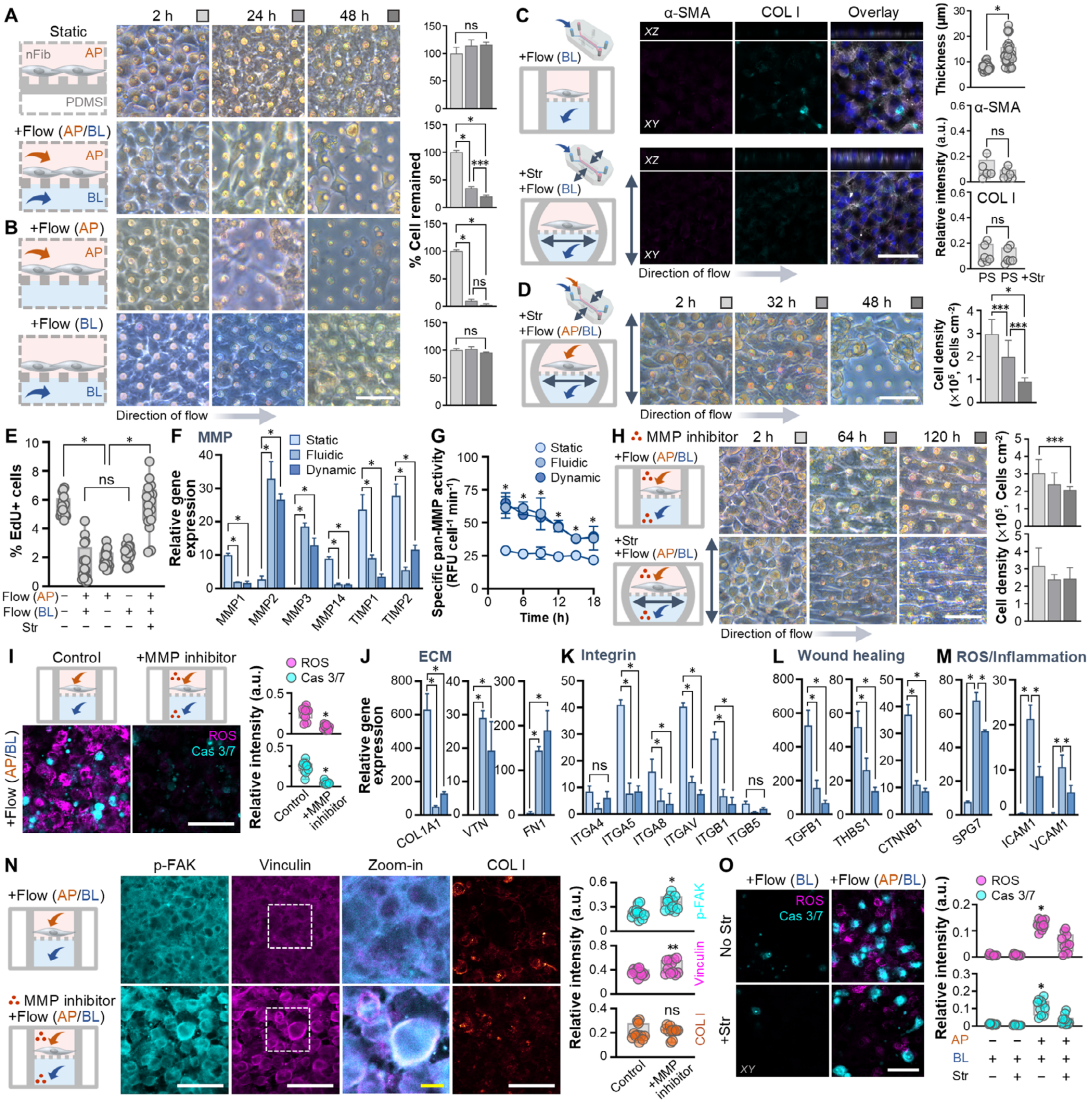
Normal intestinal fibroblasts undergo apoptotic injury under apical fluid shear stress in an MMP‐dependent manner. (A) nFib cultured on PDMS membranes under static conditions (Static) remained viable for 48 h, whereas nFib exposed to fluid flow (20 µL h^−1^) on both AP and BL microchannels (+Flow, AP/BL) in a gut‐on‐a‐chip exhibited irreversible cell death within 48 h. Phase‐contrast images were acquired at 2, 24, and 48 h. (B) nFib were hypersensitive to apical shear stress (+Flow, AP) but remained tolerant to basolateral shear stress (+Flow, BL). Quantification of remaining cells, normalized to initial cell counts at 0 h, is shown in the right panel in A and B (*n* = 5). (C) IF micrographs at 94 h demonstrated that basolateral shear stress does not induce cell death or expression of α‐SMA (magenta) or COL I (cyan), irrespective of repetitive mechanical stretching (+Str). Overlaid images include signals of F‐actin (gray) and nuclei (blue). Cell‐layer thickness (top right) and expression levels of α‐SMA and COL I (bottom right) are quantified (*n* = 3). (D) Dynamic culture conditions combining continuous fluid shear (+Flow, AP/BL) and cyclic strain (+Str) resulted in extensive nFib death independent of mechanical stretching. Phase‐contrast images were acquired at 2, 32, and 48 h to capture the initial stage, the latest time point at which an intact monolayer was still present, and the final stage following shear‐induced apoptosis, respectively. Cell numbers were quantified by nuclear counts (right panel) (*n* = 3). (E) Proliferative populations of nFib subjected to various flow conditions (AP vs. BL) and cyclic deformations (Str) were evaluated using EdU incorporation assays (*n* = 4). (F) qPCR analysis revealed changes in matrix metalloproteinase (MMP)‐associated genes (*MMP1*, *MMP2*, *MMP3*, *MMP14*, *TIMP1*, and *TIMP2*) under static, fluidic (dual flow, AP/BL), and dynamic (dual flow + cyclic stretching) conditions after 18 h (*n* = 3). (G) Pan‐MMP catalytic activity was measured under static, fluidic, and dynamic conditions using a pan‐MMP substrate [33] (*n* = 3). (H) Treatment with the pan‐MMP inhibitor (GM6001, 25 µm; red circles in schematic) rescued nFib viability under both fluidic and dynamic conditions. Phase‐contrast images were acquired at 2, 64, and 120 h to capture the initial, mid‐point, and final stages of the culture, with cell counts quantified by nuclei staining (*n* = 3). (I) Treatment of a pan‐MMP inhibitor (GM6001, 25 µm, for 18 h) significantly reduced cytoplasmic ROS generation and caspase‐3/7 activity compared to fluidic controls (20 µL h^−1^) under AP/BL flow in the gut‐on‐a‐chip. Quantification is shown in the right panels (*n* = 10). (J–M) Gene expression profiles of (J) ECM components (*COL1A1*, *VTN*, *FN1*), (K) integrins (*ITGA4*, *ITGA5*, *ITGA8*, *ITGAV*, *ITGB1*, *ITGB5*), (L) wound healing‐related genes (*TGFB1*, *THBS1*, *CTNNB1*), and (M) oxidative stress and inflammation markers (*SPG7*, *ICAM1*, *VCAM1*) (*n* = 3). (N) Addition of MMP inhibitor (GM6001, 25 µm) during dual‐flow culture of nFib preserved FAK phosphorylation, vinculin organization, and cell morphology while exerting minimal effects on COL I signal. White dashed squares indicate the location of Zoom‐in views. Quantification is shown in the right panels (*n* = 11). (O) Visualization of cytoplasmic ROS (magenta) and apoptotic cell death (cyan) using fluorogenic probes for ROS and caspase‐3/7 (Cas 3/7) activity under AP and/or BL shear stress (+Flow, BL vs. AP/BL) with or without cyclic stretching (+Str). Quantification is shown in the right panels (*n* = 3). Double arrows denote the direction of cyclic stretch (C, D, and H), and gray arrows represent medium flow direction (B–D and H). Data in box plots (3C, 3E, 3I, 3N, and 3O) show median with min‐to‐max whiskers. Data in column charts (3A, 3B, 3D, 3F‐3H, and 3J‐3 M) are presented as mean ± SEM. Statistical significance was determined using unpaired, two‐tailed Student's *t*‐test (3C), unpaired two‐tailed Student's *t*‐test (3N), one‐way ANOVA with Tukey's multiple comparisons test (3A, 3B, 3D, 3E, 3H, and 3O), or two‐way ANOVA with Sidak's multiple comparisons test (3F, 3G, and 3J‐3 M). Bars: 50 µm (white); 10 µm (yellow). ^*^ *p*<0.001, ^**^ *p*<0.01, ^***^ *p*<0.05. ns, not significant.

Next, we examined how aberrant biomechanical stimulation influences the phenotypic and molecular perturbations on nFib using a gut‐on‐a‐chip model. Exposure to apical shear stress (20 µL h^−1^) progressively induced morphological damage in nFib, resulting in significant cell detachment and apoptotic cell death over time (Figure 3A, +Flow AP/BL), whereas cells remained viable under static conditions on the same PDMS membrane (Figure 3A, Static). Notably, apical shear stress alone (+Flow AP) was sufficient to trigger cell detachment and apoptosis within 24 h (Figure 3B), while basolateral shear stress (+Flow BL; 20 µL h^−1^) caused no comparable damage at 48 h and remained non‐detrimental up to 120 h (Figure S3A). Under these conditions, BL flow did not induce upregulation of α‐SMA or COL I expression (Figure 3C, +Flow BL), even when combined with cyclic mechanical strain (Figure 3C, +Flow BL, +Str). When cyclic stretch was applied with BL flow, nFib remained viable and exhibited a significantly increased cell layer thickness (∼1.68‐fold compared to pseudostatic conditions; *p*<0.001). However, nFib cells did not display orientational alignment in response to cyclic stretch with no difference compared to BL flow alone (Figure S3B). However, when fluid shear stress was applied to both channels (+Flow AP/BL) in conjunction with cyclic stretch, cell viability was maintained only up to 32 h, after which significant damage occurred, with over 70% of nFib cells exhibiting cell death (Figure 3D). Under static conditions, the proliferative nFib population was 5.54±0.18%. However, even brief exposure (<18 h) to fluid shear stress significantly reduced proliferation (*p*<0.001), regardless of shear direction (1.90±0.15% for AP, 2.31±0.14% for BL, and 1.60±0.33% for both; Figure 3E). While cyclic strain increased proliferation to 5.55±0.42% (*p*<0.001; Figure 3E, +Flow AP/BL +Str), it was insufficient to maintain cell viability under fluid shear stress (Figure 3D). These findings confirmed that direct exposure to apical shear stress is a crucial determinant of nFib cell fate, causing detachment and atrophic damage.

Apical shear stress induced nFib cell shrinkage and detachment from the basement membrane, suggesting perturbation of genes involved in ECM regulation, cell adhesion, and stability. qPCR analyses revealed significant upregulation of matrix metalloproteinases 2 (*MMP2*) and *MMP3* and downregulation of *MMP1*, *MMP14*, tissue inhibitors of metalloproteinases 1 (*TIMP1*), and *TIMP2* under both fluidic (+Flow AP/BL) and dynamic (+Flow AP/BL +Str) conditions compared to static controls (Figure 3F). The specific MMP activity peaked at 3 h in both Fluidic and Dynamic conditions, then progressively decreased over time, whereas activity remained consistently low in static cultures regardless of the culture period (*p*<0.001; Figure 3G). This elevated MMP activity, linked to excessive ECM degradation, likely contributed to atrophic cell death [32]. When treated with a pan‐MMP inhibitor (GM6001; 25 µm), nFib cells were viable under shear stress conditions (+Flow AP/BL) for up to 120 h regardless of cyclic stretching, confirming the critical role of MMP regulation in maintaining nFib cell viability (Figure 3H; Figure S3C). Notably, treatment with a pan‐MMP inhibitor resulted in a pronounced orientational alignment under combined exposure to fluid flow and cyclic mechanical deformations as a function of time, compared to fluid flow alone (Figure S3D). Exposure of nFib cells to apical fluid shear stress for 18 h exhibited a strong signal of cytoplasmic reactive oxygen species (ROS) and caspase 3/7 (Cas 3/7)‐mediated apoptotic activity, which was significantly (*p*≤0.001) mitigated when MMP inhibitor was introduced during the microfluidic cultures (Figure 3I). Additionally, both Fluidic and Dynamic conditions led to heterogeneous ECM gene expression, with decreased collagen type I alpha 1 (*COL1A1*) and increased vitronectin (*VTN*) and fibronectin (*FN1*) levels (Figure 3J). Integrin‐associated genes, such as integrin subunit alpha 4 (*ITGA4*), *ITGA5*, *ITGA8*, *ITGAV*, integrin beta 1 (*ITGB1*), and *ITGB5*, were significantly downregulated under both Fluidic and Dynamic conditions (*p*<0.001; Figure 3K). Genes related to homeostatic wound healing processes, including transforming growth factor beta 1 (*TGFB1*), thrombospondin‐1 (*THBS1*), or catenin beta 1 (*CTNNB1*), were also markedly reduced in both Fluidic and Dynamic cultures compared to Static controls (Figure 3L). In contrast, genes associated with oxidative stress (*SPG7*) and inflammatory adhesion responses, such as intercellular adhesion molecule 1 (*ICAM1*) and vascular cell adhesion molecule 1 (*VCAM1*), were significantly elevated under both Fluidic and Dynamic conditions (Figure 3M). We found that shear stress alone induced a pronounced disruption of focal adhesion architecture, evidenced by reduced phosphorylated focal adhesion kinase (p‐FAK) levels, disorganized vinculin structures, and marked cell shrinkage preceding detachment and apoptosis (Figure 3N). Importantly, these pathological features are significantly rescued by MMP inhibition, with preservation of both p‐FAK signaling and vinculin organization despite ongoing shear, indicating that MMP activity acts upstream of focal adhesion failure. Under fluid shear stress, however, COL I signal was detected at a relatively low level in the basement region adjacent to fibroblasts, with no significant differences observed between control and GM6001‐treated conditions. Exposure of nFib cells to apical fluid shear stress for 18 h markedly increased cytoplasmic ROS and Cas 3/7‐mediated apoptotic activity (*p*<0.001) compared to pseudostatic controls (Figure 3O, +Flow BL; Figure S3E,F). Notably, the application of cyclic mechanical strain significantly attenuated both ROS generation and Cas 3/7 activity (*p*<0.001), indicating a protective effect against shear‐induced apoptosis. Together, these findings highlight fluid shear stress as a dominant biomechanical regulator of nFib viability, with an inverse relationship between apoptosis and proliferation determining mechanoresponsive cell fate.

### 2.4 Intact Epithelial Barrier Protects against Shear‐Induced Fibroblast Injury

**FIGURE 4:**
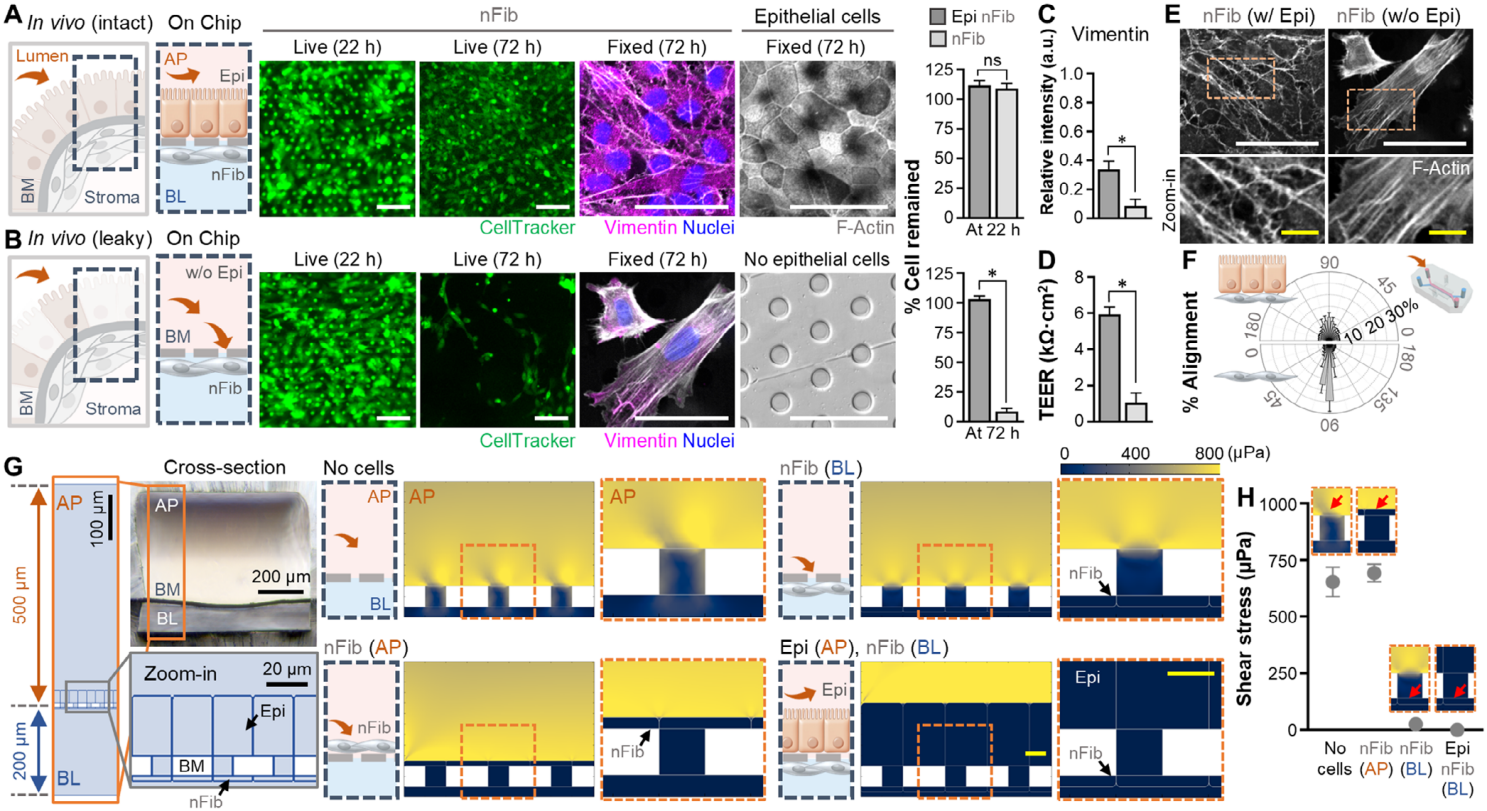
An intact intestinal epithelial barrier protects normal fibroblasts from fluid shear‐induced injury in a gut‐on‐a‐chip. (A) Schematic illustrating an intact epithelial‐fibroblast interface in vivo (left) and its in vitro reconstitution in a gut‐on‐a‐chip (right). Human intestinal epithelial cells from biopsy‐derived organoids were co‐cultured with nFib on opposite sides of a porous basement membrane (BM) to establish epithelial‐stromal compartmentalization. Apical fluid flow (100 µL h^−1^) was applied for up to 72 h. Live nFib morphology was tracked using CellTracker Green, and fixed samples at 72 h were analyzed by immunofluorescence confocal microscopy for vimentin (magenta), F‐actin (gray), and nuclei (blue). nFib numbers were quantified by nuclear counting (*n* = 3). (B) Schematic depicting impaired epithelial barrier in vivo (left) and its in vitro simulation (right), in which nFib were cultured alone on the basal side of the porous membrane. Live‐cell tracking and endpoint immunofluorescence analysis were performed as in panel A following 72 h of apical fluid flow (*n* = 3). (C) Quantification of vimentin expression based on relative fluorescence intensity from panels A and B (*n* = 9). (D) TEER measurements comparing barrier integrity between epithelial‐fibroblast co‐cultures and fibroblast mono‐cultures (*n* = 9). (E) Comparison of actin cytoskeletal organization in nFib cultured in the presence (left) or absence (right) of an epithelial layer. White dashed boxes in the upper panels denote regions shown at higher magnification below. (F) Quantification of nFib alignment (orientation index) in the presence (upper) and absence (lower) of epithelial cells, based on images shown in panel E (*n* = 8). (G) Schematics of vertical cross‐sections of gut‐on‐a‐chip microchannels and their dimension (orange boxes), highlighting the epithelial‐nFib interface around the BM (gray boxes). Culture configurations include: No cells, nFib seeded on the AP side (nFib AP), nFib seeded on the BL side (nFib BL), and epithelial cells on the AP side with nFib on the BL side (Epi AP, nFib BL). Corresponding 2D simulations visually represent fluid shear stress distributions under each configuration. (H) Quantification of fluid shear stress experienced at the nFib surface under each configuration, as indicated by red arrows in the insets (No cells, *n* = 129; nFib AP, *n* = 2,785; nFib BL, *n* = 279; Epi AP nFib BL, *n* = 1038). Data in column charts (4A‐4D, 4F, and 4H) are presented as mean ± SEM. Statistical significance was determined by unpaired, two‐tailed Student's *t*‐test (4A‐4D and 4F) or one‐way ANOVA with Tukey's multiple comparisons test (4H). Bars: 50 µm (white); 10 µm (yellow). ^*^ *p*<0.001, ^**^ *p*<0.01, ^***^ *p*<0.05; ns, not significant.

The intestinal epithelial barrier normally shields underlying fibroblasts from direct exposure to luminal fluid shear stress. We hypothesized that aberrant shear exposure could have detrimental effects on nFib. To verify this hypothesis, we employed a gut‐on‐a‐chip model to simulate either an intact mucosal barrier (epithelial‐fibroblast co‐culture; Figure 4A, schematic) or a compromised epithelial barrier (fibroblasts alone; Figure 4B, schematic) under apical fluid flow. In the intact barrier model, normal intestinal organoid‐derived epithelial cells were seeded into the apical microchannel and nFib cells into the basolateral microchannel on opposing sides of a porous membrane. Apical flow was applied at 100 µL h^−1^, 5 times higher than the previous setup, for up to 72 h to challenge the system and assess epithelial protective effect against shear‐induced fibroblast injury. Under intact barrier conditions, nFib labeled with a fluorescent dye (CellTracker green) maintained high confluency at 22 h (Figure 4A) and stable vimentin expression at 72 h (Figure 4C). In contrast, in the absence of epithelial layers, nFib exhibited marked cell loss, weakened vimentin staining, prominent actin stress fibers, indicative of cytoskeletal reorganization and injury (Figure 4B,C). Transepithelial electrical resistance (TEER) was significantly higher (*p*<0.001) in epithelial‐fibroblast co‐cultures compared to fibroblast monocultures (Figure 4D), confirming the protective role of barrier integrity. Morphological analysis further revealed that nFib under intact epithelium displayed random F‐actin organization (Figure 4E, left) and stochastic alignment (Figure 4F), whereas fibroblasts exposed directly to shear stress showed pronounced elongation and directional alignment (Figure 4E, right). These findings elucidate the critical role of the epithelial barrier in mitigating the adverse effects of luminal shear stress on underlying fibroblasts and highlight its importance in maintaining intestinal tissue homeostasis.

To characterize the influence of fluid dynamics on fibroblast responses, computational simulations were conducted to estimate fluid shear stress profiles at the cell surface, reflecting the geometry of the gut‐on‐a‐chip (Figure S4A). Four configurations were modeled: a cell‐free control (No cells), fibroblast monocultures positioned on the apical side (nFib AP) or basolateral side (nFib BL), and an epithelial‐fibroblast co‐culture (Epi AP, nFib BL). Simulations revealed the highest shear stress levels (692.06±0.15 µPa) on nFib cells directly exposed to luminal flow at the apical surface (Figure 4G,H). In contrast, shear stress was negligible when nFib cells were positioned beneath the basement membrane, aligning well with linear flow rate profiles (Figure S4B). Indeed, apical fluid transmitted through 10 µm pores exerted substantially lower shear stress (25.76±0.80 µPa) on underlying fibroblasts. However, localized fluidic eddies, identified by surface velocity fields at steady state (Figure S4C, arrows), contributed to progressive nFib cell loss on the BL side. These findings confirmed that an intact epithelial barrier is necessary and sufficient to preserve nFib viability under luminal shear conditions.

### 2.5 Longitudinal Exposure to Luminal Fluid Shear Induces the Transition of Normal Fibroblasts into Myofibroblast‐Like Cells

**FIGURE 5:**
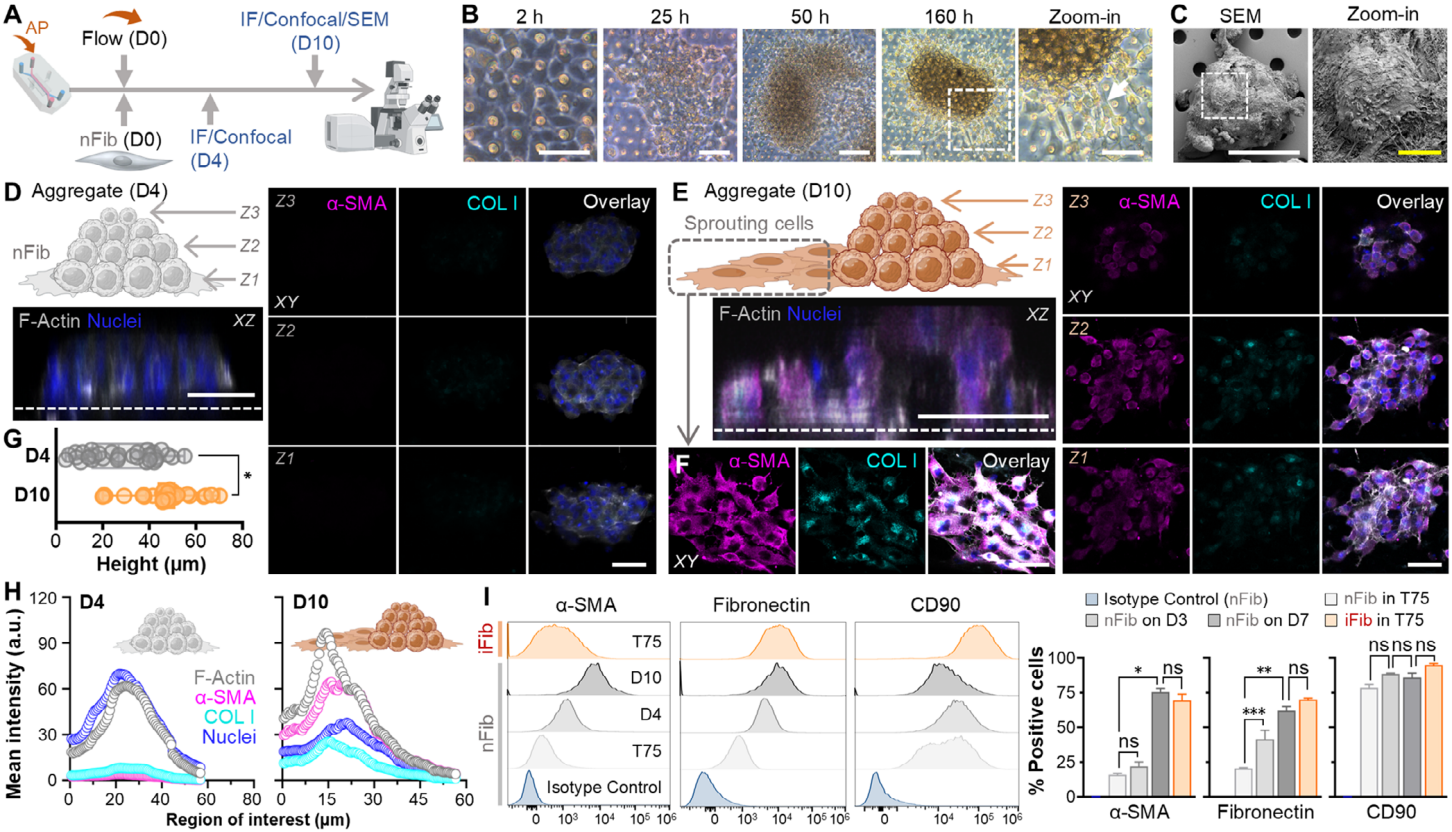
Prolonged exposure to fluid shear drives the transition of normal fibroblasts to a myofibroblast‐like state. (A) Experimental workflow demonstrating the introduction of apical flow to nFib, followed by IF confocal microscopy and scanning electron microscopy (SEM). Analyses were performed at day 0 (D0), day 4 (D4), and day 10 (D10). (B) Phase‐contrast micrographs showing progressive morphological changes in nFib exposed to apical shear stress (20 µL h^−1^) in the gut‐on‐a‐chip. The imaging time points (2, 25, 50, and 160 h) were selected at approximately day‐scale intervals to capture representative morphological changes over the course of culture. These intervals were intended to provide an observational overview of temporal progression. The dashed white square denotes the region shown at higher magnification, and a white arrow indicates sprouting cells emerging from the PDMS membrane. (C) SEM images revealing the formation of hemi‐spherical nFib aggregates (left), with a magnified view of the dashed region (right). (D) IF confocal images of aggregates at D4 showing low expression of α‐SMA (magenta) and COL I (cyan). Overlays include F‐actin (gray) and nuclei (blue). Z1‐Z3 denotes the z‐positions corresponding to XY views. (E) IF images of aggregates at D10 demonstrating increased α‐SMA and COL I expression, with corresponding XY views at Z1, Z2, and Z3. Overlays show F‐actin (gray) and nuclei (blue). (F) IF views of α‐SMA and COL I expression in sprouting nFib cells extending from aggregates onto the PDMS surface at D10. (G) Quantification of aggregate height at D4 and D10 (*n* = 33). (H) Mean fluorescence intensity profiles of α‐SMA, COL I, F‐actin, and nuclei across aggregates at D4 and D10. (I) Flow cytometry histograms comparing nFib cultured in T75 flasks, nFib from aggregates at D4 and D10, and iFib cultured in T75 flasks, assessing fibrosis‐associated markers including α‐SMA, fibronectin, and CD90. Quantification is shown in the right panel (*n* = 3). Data in column charts (5I) are presented as mean ± SEM. Data in box plots (5G) show median with min‐to‐max whiskers. Statistical significance was determined by unpaired, two‐tailed Student's *t*‐test (5G) or one‐way ANOVA with Tukey's multiple comparisons test (5I). XY: top‐down views; XZ: vertical cross‐sectional views. Bars: 50 µm (white), 10 µm (yellow). ^*^ *p*<0.001, ^**^ *p*<0.01, ^***^ *p*<0.05; ns, not significant.

As noted, when nFib cells were subjected to continuous fluid shear in a gut‐on‐a‐chip for several days (Figure 5A), the majority of nFib cells underwent apoptotic cell damage, leading to a substantial cell shrinkage within a day (Figure 5B, 25 h; Movie S3). Interestingly, a few surviving populations among dying nFib cells formed sporadic cell aggregates (Movie S4), then progressively increased their size up to several hundred micrometers under the exposure to continuous fluid shear stress (Figure 5B, 50 and 160 h). Some aggregates began sprouting cells that spread across the surface of a membrane, demonstrating remarkable robustness under fluid shear stress (Figure 5B, 160 h Zoom‐in). Scanning electron microscopy (SEM) revealed that the surface of these aggregates was covered with massive collagen‐like fibrils with multiple superposed cells underneath the fibrils (Figure 5C; Figure S5A). The aggregates harvested at days 4 (D4) and 10 (D10) showed distinct phenotypic changes, where immunofluorescence confocal microscopy revealed that D4 aggregates lacked detectable expression of α‐SMA and COL I across all vertical locations (Figure 5D). In contrast, D10 aggregates exhibited strong α‐SMA expression and mild COL I expression throughout their vertical cross‐sectional structure (Figure 5E). Notably, the sprouting populations on the membrane surface displayed the most remarkable α‐SMA expression, overlapping with actin stress fibers, indicating a transition into mechanoadaptive myofibroblast‐like cells (Figure 5F). Quantitative analysis confirmed the progressive growth of these aggregates, with the average height of D10 aggregates (46.69 ± 2.07 µm) significantly greater (*p*<0.001) than that of D4 aggregates (28.36 ± 2.57 µm) (Figure 5G). Fluorescence intensity profiles further validated the significant increase in both α‐SMA and COL I expression from D4 to D10 (Figure 5H; Figure S5B). Flow cytometry analysis also confirmed dramatic phenotypic shifts of nFib cells from the original state (nFib cells cultured in T75 flasks; Figure 5I, T75) to the transient state (nFib cells challenged to microfluidic conditions in a gut‐on‐a‐chip; Figure 5I, D4 & D10) or from the reference state (iFib cells cultured in T75 flasks; Figure 5I, iFib T75). We observed significant increases in fibrosis‐associated markers, including α‐SMA, fibronectin, and CD90 (Glycosylphosphatidylinositol‐anchored glycoprotein, also known as Thy‐1 [34]) (Figure 5I). Flow cytometry plots (Figure 5I, left) and their quantification (Figure 5I, right) showed a marked transition of nFib cells from their native state (T75) to myofibroblast‐like aggregates at D10, reminiscent of iFib phenotypes. Notably, D10 aggregates exhibited significant elevation in α‐SMA (∼4.72 folds) and fibronectin expression (∼3.02 folds) compared to nFib cells in T75 (*p*<0.001) but were not significantly different from iFib cells. Notably, CD90 expression was consistent across all cell types, reflecting its role as a general fibroblast marker. These findings collectively highlight the impact of fluid shear stress in driving the phenotypic reprogramming of normal fibroblasts into mechanoadaptive myofibroblast‐like cells, providing insights into the mechanobiological processes underlying fibrosis‐associated cellular dynamics.

### 2.6 Mechanoadaptive Fibroblasts from Shear‐Induced Aggregates Exhibit Profibrotic Traits

**FIGURE 6:**
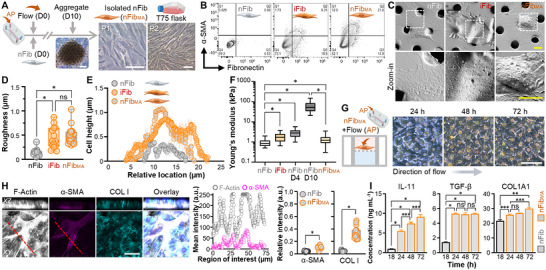
Mechanoadaptive fibroblasts derived from shear‐induced aggregates exhibit stable profibrotic phenotypes and behaviors. (A) Schematic workflow illustrating the generation of nFib aggregates under continuous apical fluid shear (20 µL h^−1^) in a gut‐on‐a‐chip, followed by isolation and expansion of mechanoadaptive fibroblasts (nFib_MA_) in T75 flasks. Representative phase contrast micrographs show nFib_MA_ morphology at passages 1 (P1) and 2 (P2) at confluence. (B) Flow cytometric comparison of nFib, iFib, and nFib_MA_ cultured on T75 flasks, highlighting expression of profibrotic markers α‐SMA and fibronectin. (C) SEM views of nFib, iFib, and nFib_MA_ cultured on PDMS membranes (top), with higher magnified views of the regions outlined by dashed boxes (bottom). (D) Quantification of surface roughness of nFib, iFib, and nFib_MA_ derived from SEM images in panel C using AFM analysis (*n* = 300). (E) Cell height measurements of nFib, iFib, and nFib_MA_ cultured statically on PDMS membranes (*n* = 15). (F) Young's modulus measurements of nFib, iFib, nFib_MA_, and nFib aggregates harvested at days 4 and 10 (D4 and D10) following microfluidic culture under apical flow (20 µL h^−1^) (*n* = 500). (G) Phase contrast micrographs of nFib_MA_ cultured in a gut‐on‐a‐chip under apical flow (20 µL h^−1^) for up to 72 h. (H) IF microscopy reveals the expression of profibrotic α‐SMA (magenta) and COL I (cyan) in nFib_MA_ cultured under fluid shear stress for 95 h in a gut‐on‐a‐chip. An overlay image includes F‐actin (gray) and nuclei (blue). Middle panels show mean fluorescence intensity profiles from line scans (red dashed lines) across F‐actin and α‐SMA signals, with quantitative analysis shown on the right (*n* = 3). (I) Quantification of secreted fibrosis‐associated markers (IL‐11, TGF‐β, and COL1A1) from nFib and nFib_MA_ cultured in a gut‐on‐a‐chip for 72 h (*n* = 3). Data in column charts (6I) are presented as mean ± SEM. Data in box plots (6D, 6F, and 6H) show median with min‐to‐max whiskers. Statistical significance was determined by one‐way ANOVA with Tukey's multiple comparisons test (6D and 6F), unpaired, two‐tailed Student's *t*‐test (6H), or two‐way ANOVA with Sidak's multiple comparisons test (6I). Bars: 50 µm (white), 10 µm (yellow). ^*^ *p*<0.001, ^**^ *p*<0.01, ^***^ *p*<0.05; ns, not significant.

To investigate the cellular and molecular characteristics of the shear‐tolerant fibroblasts within shear‐induced aggregates, D10 aggregates were enzymatically dissociated using trypsin, and the resulting cells were expanded through serial passages in T75 flasks (Figure 6A). The isolated mechanoadaptive fibroblasts (nFib_MA_) displayed a spindle‐shaped, elongated morphology reminiscent of iFib cells, with a marked anisotropic alignment. Quantitative image analysis revealed that nFib_MA_ exhibited a significantly higher orientation index compared to nFib (*p*<0.001, Figure S6A). Flow cytometry analysis confirmed that nFib_MA_ exhibited significantly elevated expression of both α‐SMA and fibronectin among CD90^+^ and vimentin^+^ cell populations, aligning closely with the expression profile of iFib (Figure 6B; Figure S6B,C). Structural assessment via SEM revealed that both iFib and nFib_MA_ had roughened surfaces with multiple microvilli‐like protrusions, a stark contrast to the smoother morphology of nFib (Figure 6C). Additionally, atomic force microscopy (AFM) quantitatively confirmed that both surface roughness (Figure 6D) and average cell height (Figure 6E) were significantly elevated (*p*<0.001) in both iFib and nFib_MA_ compared to nFib, with no substantial distinction between iFib and nFib_MA_. Moreover, AFM‐based stiffness measurements confirmed that Young's modulus values of iFib, nFib_MA_, D4, and D10 aggregates were significantly higher than nFib, indicating a progressive stiffening response to mechanobiological stress (Figure 6F).

To further examine the behavior of nFib_MA_ under sustained fluid shear stress, we cultured them within a gut‐on‐a‐chip and subjected them to continuous microfluidic flow at 20 µL h^−1^ for 72 h. Under these conditions, nFib_MA_ remained stably attached with no appreciable cell loss (Figure 6G). Confocal IF imaging revealed robust α‐SMA expression that closely aligned with actin stress fibers, alongside a marked increase in COL I deposition compared to nFib (Figure 6H). Notably, IL‐11 secretion increased over time, whereas TGF‐β and COL1A1 levels remained relatively stable or exhibited more modest temporal changes (Figure 6I). However, when nFib were subjected to fluid shear in the presence of exogenous IL‐11 and TGF‐β (10 ng mL^−1^ each), at concentrations comparable to those measured in nFib_MA_, they did not acquire the mechanoadaptive, shear‐tolerant phenotype observed in Figure 6G. Instead, their response remained indistinguishable from flow‐alone conditions, undergoing shear‐induced apoptosis and forming aggregates (Figure S6D), suggesting that IL‐11 and TGF‐β function predominantly as secondary amplifiers of profibrotic signaling rather than primary initiators of mechanoadaptation in normal fibroblasts.

## 3 Discussion

Intestinal fibrosis is a major pathological complication of IBD, yet its initiating mechanisms and regulatory pathways remain insufficiently understood. Emerging evidence suggests that mucosal damage serves as the primary site for the onset of fibrosis, implicating mucosa‐associated cells, such as intestinal epithelial cells and fibroblasts, and microenvironmental factors, including mechanostimulatory forces, as critical modulators of early profibrotic processes [3, 35, 36]. However, the absence of physiologically relevant human models has limited mechanistic investigations into how the initial fibrogenic microenvironment drives pathological progression.

In this study, we leveraged a microengineered human gut‐on‐a‐chip platform as a mechanistic discovery tool to recreate the mechanically dynamic intestinal microenvironment and elucidate its role in initiating early fibrotic events. By establishing fibroblast cultures under conditions of either intact or disrupted epithelial barriers, mimicking pathophysiological mucosal states in IBD, we were able to interrogate stromal responses to precisely controlled luminal flow and peristalsis‐like mechanical deformations. Unlike conventional static cultures, which omit the intrinsic peristaltic forces and hydrodynamic cues of the human intestine, this bioengineered MPS enables modulation of mechanotransductive signaling within a human‐relevant context. Within this conceptual framework, our findings reveal a previously unrecognized, immune‐independent, biomechanics‐driven axis of fibroblast activation with direct implications for the earliest stages of intestinal fibrosis in IBD. Our findings establish a conceptual paradigm in which mechanical forces, rather than inflammatory mediators alone, function as primary determinants of fibroblast fate during disease initiation. This concept reframes fibrosis as a mechanobiological process that can precede and operate independently of overt inflammation. This mechanistic insight not only clarifies a missing dimension in fibrosis pathogenesis but also provides a foundation for systematically interrogating upstream genetic, epigenetic, and biophysical regulators of fibrotic remodeling and for uncovering mechanotransduction‐based therapeutic targets for anti‐fibrotic intervention.

The modular design of this model offers the unique advantage of independently investigating the effect of fluid shear stress and cyclic mechanical strain, enabling the examination of their respective contributions in fibroblast activation and cell fate. Notably, this strategy has been challenging to implement in animal models and human clinical studies. A key innovation of this platform is its ability to selectively reproduce mechanobiological complexity. In vivo, the intestinal microenvironment is highly complex, with SMCs [37] contributing both mechanical forces and biochemical signals that regulate fibrosis [38]. Because SMCs not only generate contractile strain but also secrete fibrogenic factors, it is difficult to isolate the effects of mechanical strain from molecular signaling in animal models. The gut‐on‐a‐chip system overcomes this challenge by recreating peristalsis‐like mechanical deformations without SMCs, enabling mechanistic dissection of the impact of cyclic strain on fibroblast behavior. This physiologically relevant, yet reductionist, model provides a powerful strategy in a controlled environment for exploring how mechanical forces drive early fibrotic processes, insights that are difficult to obtain from conventional in vivo models.

When utilizing patient‐derived fibroblasts, it is essential to ensure that their disease‐intrinsic characteristics are stably maintained in vitro. Most previous studies on fibroblast behavior in fibrosis have focused on responses to ECM remodeling and tissue mechanoregulation, particularly matrix stiffness [39, 40]. In this context, we observed distinct morphological differences between nFib and iFib in response to varying substrate stiffness across different culture formats. AFM measurements of substrate Young's modulus aligned well with previously reported values: T‐flask (∼3 GPa) [41], polyester nanoporous membrane (<2 GPa) [42], and PDMS membrane (<1 MPa) [43]. Notably, the substrates used in this study, including an elastic PDMS membrane, exhibit significantly higher stiffness compared to in vitro hydrogels (∼0.1 kPa) [44] and in vivo IBD tissue (∼1 kPa) [45], a key factor to consider when interpreting mechanobiological responses. Consistent with prior observations, stiffer substrates promoted fibroblast elongation and alignment [46] and induced α‐SMA expression in iFib, a hallmark of myofibroblast differentiation. In contrast, nFib exhibited no detectable α‐SMA expression under identical conditions. This distinct mechanophenotypic behavior was consistently maintained across all culture formats, underscoring the reliability of patient‐derived fibroblasts for studying disease‐relevant mechanobiological responses.

Dysregulated bowel motility is one of the characteristics of IBD, yet its influence on early fibrotic remodeling and the underlying cellular mechanisms remains insufficiently understood. Our microfluidic and stretchable gut‐on‐a‐chip replicates physiologically relevant mechanical cues, in contrast to static cultures, which lack bowel motility and are mechanically abnormal. We found that fluid shear stress promoted hypertrophy in iFib, increased fibroblast layer thickness, and enhanced myofibroblast‐like activation, as indicated by increased α‐SMA, TGF‐β, and IL‐11. In contrast, nFib were highly sensitive to the same mechanical cues, exhibiting limited activation and instead undergoing significant cell death. These results demonstrate that iFib exhibit elevated mechanosensitivity, undergoing cytoskeletal reorganization and activating profibrotic signaling pathways in response to shear stress and cyclic mechanical strain. Notably, the fibrotic response was further exacerbated when cyclic stretch was combined with fluid flow, driving myofibroblast‐like activation. In contrast, nFib exhibited minimal responses to mechanical strain, indicating that iFib cells, with their predisposition to mechanically dynamic milieus, are more responsive to dynamic mechanical forces, thereby accelerating early fibrotic progression. These findings emphasize the necessity for physiologically dynamic models to effectively investigate the biomechanical drivers of intestinal fibrosis in IBD.

We experimentally demonstrated that nFib cells are highly susceptible to fluid shear stress, particularly when applied apically. This stimulation results in atrophic cell detachment followed by apoptotic cell death within approximately 24 h, even at very low shear stress (∼0.00133 dyne cm^−2^). This hyper‐sensitivity to fluid shear stress was consistent across different culture substrates and ECM coating conditions. Histologically, intestinal fibroblasts are localized beneath the epithelial barrier, where in healthy intestinal mucosa, they experience only minimal interstitial fluid shear in the sub‐epithelial area or lamina propria. Our experimental model, therefore, replicated a pathological condition in which a disrupted epithelial layer mimics disease‐associated sites (e.g., mucosal damage in IBD), within a controlled microphysiological environment. In vivo, epithelial barrier impairment triggers homeostatic processes that accompany wound healing, including myofibroblast activation through mechanical cues (e.g., cytoskeleton reorganization into stress fibers [47]), biochemical signaling (e.g., TGF‐β release, ECM deposition [48]), and functional differentiation (e.g., α‐SMA expression [49]). In this study, we reproducibly replicated these pathophysiological processes in vitro using iFib cells derived from a UC patient in the gut‐on‐a‐chip model. In contrast, nFib cells from a healthy donor exhibited extreme sensitivity to prolonged fluid shear stress, resulting in apoptotic cell death within 24 h. As such, we conducted short‐term (up to 18 h) fluid shear stress exposure, followed by comprehensive morphological, transcriptomic, and cellular assessments.

Physiological measurements consistently show that shear stress along the intestinal epithelial surface is generally very low (∼0.002–0.08 dyne cm^−2^), a range supported by multiple hydrodynamic analyses [22, 50, 51, 52]. Reassessment of human intestinal flow using reported fasting and fed flow rates in the jejunum and ileum, luminal viscosities of 0.007–0.01 g cm^−1^·s^−1^, and typical intestinal diameter (∼2.5 cm) produced shear values of 0.001–0.008 dyne cm^−2^, confirming that steady‐state epithelial shear resides in the 10^−3^ dyne cm^−2^ regime. In vivo, however, shear stress varies considerably: differences in mucus viscosity, the presence of a thin hydrated slip layer, heterogeneous bolus rheology in luminal contents, and the predominance of segmental mixing contractions, accounting for nearly 80% of intestinal motility, which together generate highly dynamic shear fields. Computational and experimental studies further demonstrate that brief, localized shear spikes of 1–5 dyne cm^−2^ can occur during bolus transport or vigorous mixing, while pathological changes such as mucosal ulceration, epithelial loss, or luminal narrowing in CD can substantially alter local flow and the shear transmitted to the stromal surface [53, 54]. Within this physiological and disease‐informed framework, we selected a shear regime of 0.0013–0.0067 dyne cm^−2^ (at 20–100 µL h^−1^) to model the impact of low‐magnitude shear to intestinal fibroblasts that becomes relevant when epithelial barrier integrity is compromised, exposing the lamina propria to luminal flow. This range approximates the shear expected during early mucosal injury, enables sensitive assessment of fibroblast vulnerability to minimal mechanical cues, and avoids artificially high shear forces that could obscure mechanoadaptive behaviors. Notably, even these low shear stresses elicited marked mechanosensitivity in nFib, inducing rapid apoptosis and promoting the development of stiff mechanoadaptive aggregates, underscoring both the physiological relevance of the chosen shear regime and its suitability for modeling early stromal events that contribute to normal intestinal fibrosis.

Cyclic stretching is known to promote fibroblast proliferation through mechanosensitive pathways such as YAP/TAZ signaling [55] or integrin‐FAK signaling [56], a finding consistent with our results (Figure 3E). However, despite this proliferative response, mechanical deformation alone was insufficient to mitigate the shear‐induced apoptotic damage in nFib cells. To investigate the underlying molecular mechanisms, we first examined the expression of MMP‐associated genes and their catalytic activity. When nFib cells were subjected to apical fluid shear stress for 18 h, we observed a significant upregulation of *MMP2* and *MMP3*, alongside a downregulation of their antagonists, including *TIMP1* and *TIMP2*. This transcriptomic alteration resulted in a marked increase in pan‐MMP catalytic activity under microfluidic conditions, whereas activity in static controls remained largely unchanged regardless of culture duration. We hypothesized that the upregulation of MMPs in response to shear stress leads to the compromised focal adhesion signaling as well as degradation of ECM components, such as collagen and fibronectin, weakening the structural support for fibroblasts. This degradation reduces focal adhesion stability, which can result in detachment‐induced apoptosis (anoikis) or cellular damage [57]. MMP‐mediated ECM degradation disrupts the anchoring points necessary for efficient force transduction. Without a stable ECM, fibroblasts are unable to properly polarize their cytoskeleton in response to cyclic strain, preventing alignment [58, 59]. This hypothesis was further validated by the use of an MMP inhibitor (GM6001), which preserved ECM integrity by inhibiting excessive ECM degradation. With an intact matrix, fibroblasts maintained stable focal adhesions, enabling proper mechanotransduction. Under these conditions, fibroblasts effectively sensed and adapted to mechanical stimuli, aligning their actin filaments and microtubules perpendicular to the direction of cyclic stretching.

Under dynamic shear stress and strain, nFib cells undergo extensive ECM remodeling, characterized by increased vitronectin (*VTN*) expression and reduced integrin levels (*ITGA5*, *ITGA8*, *ITGAV*, *ITGB1*), suggesting a decoupling of ECM production from cell adhesion. *VTN* upregulation can serve as a protective ECM‐stabilizing response [60], while integrin downregulation suppresses mechanotransduction, potentially shifting fibroblasts toward a quiescent or apoptotic state [61]. This balance could mitigate excessive ECM deposition and fibrosis by reducing TGF‐β signaling but may also impair fibroblast adhesion, migration, and mucosal healing, contributing to chronic tissue damage in IBD [62]. The downregulation of *COL1A1* suggests reduced matrix rigidity and fibroblast activation, potentially mediated by inhibition of TGF‐β (*TGFB1*) and Wnt/β‐catenin (*CTNNB1*) signaling. Meanwhile, fibronectin (*FN1*) upregulation under dynamic conditions may be a compensatory mechanism to restore ECM integrity and cell adhesion [63]. Thrombospondin‐1 (*THBS1*), a key activator of latent TGF‐β and driver of fibrosis [64], is significantly downregulated under mechanical stimulation, reducing TGF‐β activation and shifting fibroblasts toward a less fibrotic state. These findings highlight the anti‐fibrotic potential of mechanobiological cues in regulating ECM homeostasis and suggest *THBS1* as a therapeutic target for intestinal fibrosis in IBD. Additionally, spastic paraplegia 7 (*SPG7*), a mitochondrial matrix protein involved in the regulation of mitochondrial permeability transition pore (mPTP), plays a key role in oxidative stress and apoptosis by facilitating prolonged mPTP opening, leading to cytochrome c release and subsequent caspase activation [65]. We found that mechanical stress under flow conditions has been shown to increase mitochondrial activity and ROS production in cells with elevated *SPG7* expression, correlating with heightened oxidative stress observed in fibrotic tissues. In our model, fluidic flow alone induced the highest upregulation of *SPG7*, whereas the addition of cyclic mechanical strain significantly suppressed its expression. This pattern of *SPG7* regulation corresponded with changes in inflammatory gene expression (*ICAM1*, *VCAM1*), cytoplasmic ROS levels, and Cas 3/7 activity, further emphasizing the critical role of mechanical microenvironments in modulating fibroblast oxidative stress responses and inflammatory activation.

Our mechanistic analyses indicate that shear‐induced MMP activation plays a pivotal role in disrupting the focal adhesion‐integrin‐ECM axis, thereby promoting fibroblast stress and death under fluid flow. Exposure to shear stress induces elevated oxidative stress, focal adhesion disassembly, and apoptosis, as evidenced by increased ROS generation and Cas 3/7 activation, along with reduced FAK phosphorylation and disorganized vinculin structures. Importantly, global MMP inhibition robustly protects fibroblasts from ROS accumulation and apoptotic death while preserving focal adhesion signaling despite ongoing shear. Because focal adhesions form only when integrins are productively engaged with the ECM, restoration of p‐FAK signaling and vinculin organization provides functional evidence that MMP inhibition maintains effective integrin‐ECM coupling. In this framework, focal adhesion readouts serve as integrative and mechanistically proximal indicators of ECM integrity, capturing the downstream consequences of MMP‐mediated extracellular remodeling on adhesion signaling. Together, these findings establish MMP activity as a key upstream regulator linking shear stress to oxidative injury, focal adhesion failure, and fibroblast fate, and identify the focal adhesion‐integrin‐ECM axis as a central mechanobiological pathway governing stromal cell survival in the dynamic intestinal microenvironment.

Our findings confirm that an intact epithelial barrier is essential for protecting shear‐sensitive normal fibroblasts. When this barrier is compromised, nFib cells experience severe damage and undergo apoptosis. Interestingly, a subset of fibroblasts exhibited persistence under shear stress, forming atypical cellular aggregates. Ultrastructural SEM analysis revealed that these aggregates consist of multiple fibroblasts embedded within a dense, complex fibrillar network, a previously unreported, unique morphological feature. This dome‐shaped aggregation and intricate fibrillar microstructure may create a protective niche, enabling fibroblasts tolerant to shear stress and transition into mechanoadaptive myofibroblast‐like cells. This process likely reflects localized excessive ECM deposition, potentially representing an early stage of fibrosis in IBD. AFM analysis demonstrated that the stiffness of these aggregates on day 10 closely matched the tissue stiffness (∼10–15 kPa) observed in CD patients with severe stricture formation, whereas normal gut shows ≤3 kPa^29^, suggesting that shear‐induced stiff 3D aggregate formation may illustrate pathological manifestation of fibrotic remodeling relevant to stricture formation in CD.

Mechanoadaptive fibroblasts (nFib_MA_) isolated from these aggregates exhibited key myofibroblast‐like characteristics, including increased hypertrophy, rough cell surfaces, elevated stiffness, and α‐SMA expression aligned with actin stress fibers. Notably, nFib cells did not express detectable α‐SMA under any conditions, including variations in culture substrate, mechanical stimulation, short‐term fluid shear stress, or exposure to profibrotic factors such as IL‐11 or TGF‐β. Flow cytometry further confirmed that nFib_MA_ cells express fibrotic markers comparable to iFib cells, reminiscent of the activated myofibroblast populations that accumulate near ulcerated lesion [66]. These findings indicate that prolonged mechanodynamic stimulation induces a robust reprogramming of fibroblast phenotype. The term of “mechanoadaptive fibroblasts” was selected to illustrate i) the subset of normal fibroblasts that survive continuous fluid shear exposure, ii) the emergence of profibrotic phenotypes accompanied by complex fibrillar microarchitectures within the aggregates, and iii) the associated alterations in morphology, molecular signatures, and functional behavior. Notably, the morphological and molecular characteristics of nFib_MA_ remained unchanged across repeated passages, even after mechanical cues were ceased. This stability suggests an underlying regulatory shift and highlights the need for further studies to determine whether this experimentally observed persistence reflects underlying genomic, transcriptomic, or epigenomic alterations.

Our findings also clarify the role of IL‐11 and TGF‐β in nFib_MA_ transitions. Although nFib constitutively secretes both cytokines across all culture formats, neither endogenous production nor exogenous supplementation was sufficient to initiate the nFib‐to‐nFib_MA_ conversion. Fluid shear in the presence of IL‐11/TGF‐β failed to rescue cells from shear‐induced apoptosis or promote the acquisition of mechanoadaptive features, and prolonged cytokine exposure under static conditions did not induce α‐SMA production, cytoskeletal remodeling, or the shear‐tolerant phenotype characteristic of nFib_MA_. These data support a mechanistic hierarchy in which biomechanical cues, specifically fluid shear combined with MMP‐dependent matrix remodeling, serve as the primary determinants of survival vs. mechanoadaptation, while IL‐11 and TGF‐β rise only after this transition and function as secondary profibrotic amplifiers rather than primary initiators. This distinction emphasizes that cytokine‐driven pathways contribute to downstream matrix accumulation and niche stabilization, but the decisive trigger for nFib_MA_ emergence lies in the biomechanical microenvironment rather than in canonical profibrotic cytokine signaling.

Although shear‐ and strain‐driven mechanobiology has been extensively elucidated in vascular systems, the corresponding principles governing fibrotic mechanobiology in the intestine remain largely unexplored. In contrast to endothelial cells, intestinal fibroblasts are physiologically shielded from luminal flow by an intact epithelial barrier and a mucus layer; consequently, their exposure to shear stress during barrier disruption provokes distinct cellular responses. Here, we delineate gut‐specific stromal behaviors that are not anticipated by vascular paradigms: shear‐naïve normal fibroblasts predominantly undergo pronounced, MMP‐dependent apoptosis rather than adaptive cytoskeletal alignment, while only a minor surviving subpopulation acquires shear tolerance and transitions into a profibrotic, mechanoadaptive state. This transition is accompanied by niche‐like ECM remodeling and progressive matrix stiffening. Collectively, these findings highlight fundamental differences in microenvironmental context, shear magnitude, and phenotypic outcomes between intestinal and vascular tissues. Accordingly, our work establishes a gut‐specific mechanobiological framework for understanding how shear and strain govern fibroblast fate during mucosal injury and fibrotic progression, processes that cannot be inferred from vascular mechanobiology alone.

Despite the interesting findings, our study has several limitations. First, we primarily relied on a limited number of fibroblast sources, necessitating validation with additional normal intestinal fibroblast populations to confirm the shear‐induced transition. Given the significant heterogeneity of fibroblasts within the intestine, further investigation into the subpopulations of patient‐derived fibroblasts would be valuable to enhance the translational relevance of these findings. Second, our model included only two mucosal cell types, epithelium and fibroblast. To better replicate the mucosal microenvironment and elucidate intercellular crosstalk in early fibrogenesis, future studies should incorporate additional cell types such as smooth muscle cells, enteric neurons, intestinal microvascular endothelial cells, and gut microbiota. Third, while this study highlights the role of biomechanical cues as inducers of fibrosis, their potential suppressive effects were not extensively explored. Further investigations should address this aspect to provide a more comprehensive understanding of fibrosis regulation. Fourth, we intentionally excluded exogenous TNF‐α and other inflammatory cytokines to isolate the biomechanical determinants of fibroblast fate. This reductionist approach was necessary to avoid confounding effects, as TNF‐α simultaneously disrupts epithelial junctions and directly activates fibroblasts, obscuring the specific contribution of shear‐barrier interactions. However, TNF‐α, and likely additional inflammatory mediators, plays a central role in IBD pathophysiology. Future studies will therefore be required to integrate these cytokines with the mechanobiological framework established here to determine how inflammatory cues (e.g., inflammatory cytokines or IBD patient plasma) and biomechanical forces converge to shape fibroblast activation and fibrotic progression. Finally, our gut‐on‐a‐chip model employs a PDMS‐based membrane, which differs in stiffness from the native basement membrane and surrounding ECM. Incorporating patient‐derived cellular and acellular components to construct a pathophysiologically relevant basement membrane and ECM composition could improve the fidelity and relevance of disease modeling.

## 4 Conclusion

This study demonstrated that fluid shear stress is a central biomechanical driver of fibroblast fate and profibrotic remodeling, while epithelial barrier integrity serves as a critical safeguard against pathological mechanoadaptation. Using a physiodynamic human gut‐on‐a‐chip model, we revealed how mechanical forces and barrier dysfunction converge to trigger disease‐specific fibroblast responses that are otherwise difficult to capture in clinical settings. By integrating mechanical cues, cellular interactions, and patient‐derived samples, this system uncovered the early triggers of intestinal fibrosis and provides a translationally relevant platform for identifying therapeutic targets. These findings advance our understanding of intestinal mechanobiology and establish the gut‐on‐a‐chip as a versatile tool for guiding translational precision medicine in inflammatory bowel disease and fibrotic disorders.

## 5 Experimental Section

### 5.1 Device Fabrication

The gut‐on‐a‐chip device was fabricated using standard photolithography and soft lithography techniques, as previously described [20, 22]. Polydimethylsiloxane (PDMS; Sylgard 184, Dow Corning) was prepared by mixing the base elastomer and curing agent at a 10:1 (wt wt^−1^) ratio, then cast into SU‐8‐patterned silicon molds to form the upper and lower microchannel layers. After curing at 60°C for 6 h, the PDMS layers were demolded to yield channels with dimensions of 1 mm width, 1 cm length, and heights of 500 µm (upper channel) and 200 µm (lower channel), respectively. A porous, elastic PDMS membrane (10 µm pore diameter, 25 µm center‐to‐center spacing, 10 µm thickness; prepared with a 10:1 base‐to‐curing‐agent ratio) was fabricated following established protocols [20] and placed between the two channel layers to mimic the intestinal basement membrane. Each microchannel was connected to programmable syringe pumps via silicone tubing (inner diameter: 1/32“, outer diameter: 3/32”; Tygon 3350, Beaverton) and 90°‐bent blunt‐end stainless steel needles (18G; Kimble Chase) for fluidic control. Two side chambers flanking the central cell culture channel were integrated into the device to enable the application of cyclic mechanical strain.

### 5.2 Cell Cultures

Primary human intestinal fibroblasts were used to model healthy and inflammation‐associated stromal responses. Normal intestinal fibroblasts (nFib; cat. no. 2920; ScienCell) and patient‐derived inflammation‐associated fibroblasts (iFib) isolated from the inflamed colonic tissue from a patient with ulcerative colitis were cultured in a fibroblast culture medium (Dulbecco's Modified Eagle Medium, DMEM; Gibco) supplemented with 20% (vol vol^−1^) heat‐inactivated fetal bovine serum (FBS; Gibco), 1% (wt vol^−1^) L‐glutamine, and antibiotics (100 U mL^−1^ Penicillin and 100 µg mL^−1^ Streptomycin; Gibco). Cells (passage number <6) were maintained in 75 cm^2^ tissue culture flasks at 37°C in a humidified 5% CO_2_ incubator and passaged upon reaching ∼90% confluency.

The iFib cells were isolated from surgically resected de‐identified colonic specimens of a UC patient with severe inflammatory injury (S‐241113‐00981) under an approved protocol by the Institutional Review Board (IRB; 06–050) of the Cleveland Clinic Tissue Center. A consent statement was not required for this specimen, as the tissue was procured from discarded surgical material in accordance with IRB guidelines. Tissues were rinsed in phosphate‐buffered saline (PBS, Ca^2+^ and Mg^2+^ free; Gibco), minced into fragments (<0.2 mm), and enzymatically digested in Hank's Balanced Salt Solution (HBSS; Gibco) containing 10% (v v^−1^) FBS, antibiotics cocktail (100 units mL^−1^ Penicillin, 100 µg mL^−1^ Streptomycin, and 0.25 µg mL^−1^ Amphotericin B; cat. no. 10‐378‐016; Gibco), and a cocktail of collagenases: type I (100 U mL^−1^; cat. no. 1639), type II (100 U mL^−1^; cat. no. 1764), and type IV (100 U mL^−1^; cat. no. C5138; all from Sigma–Aldrich). Digestion was carried out at 37°C for up to 3 h in a shaking water bath. The resulting cell suspension was triturated gently, filtered through a 70 µm cell strainer (Celltreat Scientific Products), and centrifuged (300×*g* for 10 min, at 4°C) to isolate fibroblasts. The purified iFib population was expanded and used for downstream experiments.

Normal human intestinal organoids were derived from deidentified biopsy samples obtained from a healthy donor (62‐year‐old Caucasian male) under an approved IRB (2017‐06‐0114) from Dell Medical School, The University of Texas at Austin. Informed consent was obtained from the donor prior to tissue collection. Biopsy specimens were acquired from the right colon during a routine endoscopic examination performed for clinical evaluation. Following tissue processing, intestinal crypts were isolated and embedded in Matrigel (Corning; cat. no. CB‐40234) and maintained in a defined organoid culture medium as previously described [26]. Organoids were cultured at 37°C in a humidified 5% CO_2_ incubator, with medium changes every other day. Passaging was performed every 7 days to maintain long‐term culture and proliferation.

### 5.3 Microfluidic Cultures

Prior to cell seeding, the gut‐on‐a‐chip devices were sterilized by sequentially introducing 70% (vol vol^−1^) ethanol into the microchannels, followed by incubation at 60°C for 6 h and UV‐ozone treatment using a UVO Cleaner 342 (Jelight Company Inc.) for 40 min. Surface functionalization was performed to enhance ECM adhesion by incubating the microchannels with 1% (wt vol^−1^) polyethyleneimine (Sigma‐Aldrich, cat. no. 408700) for 10 min, followed by rinsing with sterile deionized water. Subsequently, 0.1% (vol vol^−1^) glutaraldehyde (Electron Microscopy Sciences, cat. no. 16320) was introduced for 30 min at room temperature, after which the channels were thoroughly flushed with deionized water, then completely dried. To prepare the ECM coating, an ice‐cold solution containing collagen I (0.03 mg mL^−1^; Gibco, cat. no. A10483‐01) and Matrigel (0.3 mg mL^−1^; Corning) was injected into the channels and incubated at 37°C for 1 h to promote matrix adsorption. Prior to fibroblast seeding, the upper microchannel was perfused with fibroblast culture medium at 20 µL h^−1^ for 12 h to precondition the matrix‐coated surface. For static control experiments, a flat PDMS membrane was placed in a glass‐bottom dish (Matsunami Glass Ind.; cat. no. D1113OH), sterilized with 70% ethanol, dried at 60°C for 6 h, and treated with UV‐ozone for 40 min, then followed the identical protocol for surface functionalization. For Transwell‐based static cultures, nanoporous polyester membrane inserts (pore size: 0.4 µm; Corning) were subjected to UV‐ozone treatment for 40 min to enhance surface reactivity prior to ECM coating.

Dissociated fibroblasts (1 × 10^7^ cells mL^−1^) were then introduced into the upper microchannel and allowed to adhere under static conditions for 2 h in a humidified 37°C incubator with 5% CO_2_. After attachment, culture medium was perfused into the upper and/or lower microchannels at a constant flow rate of 20 µL h^−1^. To recapitulate peristalsis‐like mechanical stimulation, cyclic deformation (10% strain, 0.15 Hz frequency) was applied via vacuum chambers interfaced with a computer‐controlled tension system (FX‐6000 Tension System with FlexLink Controller; Flexcell International Corporation). The negative pressure cycles deformed the elastic PDMS membrane, thereby transmitting dynamic strain to the fibroblast or epithelial cell layers cultured atop the membrane. To establish a dual‐sided co‐culture of iFib on both sides of a porous membrane, dissociated iFib cells (1 × 10^7^ cells mL^−1^) were first introduced into the upper microchannel and incubated under static conditions for 2 h at 37°C in a humidified CO_2_ incubator. The device setup was then gently inverted, and the same iFib suspension was introduced into the opposing (now upper) microchannel, followed by a second 2 h static incubation to allow cell attachment. After ensuring iFib adhesion to both sides of the membrane, the device was returned to its upright orientation, and continuous perfusion of fibroblast culture medium was initiated at 20 µL h^−1^. For MMP inhibition experiments, the broad‐spectrum MMP inhibitor GM6001 (25 µm; Calbiochem) was added to the fibroblast culture medium during microfluidic perfusion. For static control experiments, fibroblasts (1 × 10^7^ cells mL^−1^) were seeded either onto ECM‐coated PDMS membranes placed in glass‐bottom dishes or onto nanoporous membrane inserts and cultured under standard incubator conditions (37°C, 5% CO_2_) for 5 days. To evaluate the effects of profibrotic cytokines on nFib growth in the gut‐on‐a‐chip, medium supplemented with IL‐11 and TGF‐β (final concentration, 10 ng mL^−1^ each) was perfused through the upper microchannel at 20 µL h^−1^ for 48 h in the absence of cyclic stretch. Fibroblast morphology was monitored over time using phase‐contrast microscopy.

Culture and mechanical stimulation durations were determined based on condition‐specific cellular responses and biologically relevant endpoints. For baseline characterization (Figure 1), imaging was continued until fibroblasts reached a reproducible, fully confluent, and morphologically stable monolayer (∼120 h), which was designated as the endpoint. For iFib alignment studies (Figure 2), time points were selected to resolve the kinetics of stretch‐induced alignment; although ±Flow conditions remained stable up to 120 h, a robust and stable alignment phenotype under ±Flow ±Str emerged earlier (∼93 h; 69 h of stretch), and this earlier time point was chosen to capture the establishment of alignment rather than its terminal state. For nFib shear sensitivity experiments (Figure 3), durations were constrained by rapid shear‐induced vulnerability, with morphological deterioration initiating at 18–24 h and becoming pronounced by ∼48 h; accordingly, 32 h was selected as the latest time point permitting reliable assessment of an intact monolayer under unprotected conditions. In contrast, under MMP inhibition, nFib stability was maintained under shear up to 120 h, with 64 h selected as a representative intermediate time point, and full temporal progression provided in the supplementary data. For Figure 5, imaging time points (2, 25, 50, and 160 h) were selected at approximately day‐scale intervals to capture representative morphological progression, serving an observational rather than mechanistically defined purpose.

To perform epithelial‐fibroblast co‐cultures, the gut‐on‐a‐chip device was preconditioned by perfusing an organoid culture medium through the upper microchannel and a fibroblast culture medium through the lower microchannel for 6 h at 20 µL h^−1^. Dissociated normal human intestinal epithelial organoid cells (∼1 × 10^7^ cells mL^−1^; passage number under 5) were seeded into the ECM‐coated upper channel and incubated under static conditions for 2 h to allow for attachment. Subsequently, dissociated fibroblasts were introduced into the lower channel (preconditioned with a fibroblast medium), and the device was inverted and incubated for an additional 2 h at 37°C. The chip was then restored to its original orientation, and continuous medium perfusion was resumed through both channels at 20 µL h^−1^.

### 5.4 Isolation of Mechanoadaptive Intestinal Fibroblasts

Normal intestinal fibroblasts (nFib) cultured under continuous apical shear flow (20 µL h^−1^) in a gut‐on‐a‐chip exhibited extensive apoptotic detachment within 24 h, with a residual population forming multicellular aggregates enriched in fibrillar structures. On day 10, aggregates were enzymatically dissociated using 0.25% (wt vol^−1^) Trypsin‐EDTA solution (Gibco; cat. no. 25200072) for 5 min at 37°C. Dissociated cells were collected, filtered through a 70 µm strainer, and resuspended in a fibroblast culture medium. The isolated mechanoadaptive fibroblasts (nFib_MA_) were expanded under standard conditions (37°C, 5% CO_2_) and, upon serial passaging, developed distinct morphological and phenotypic traits consistent with a myofibroblast‐like state.

### 5.5 Morphological Analysis

Fibroblast morphology was monitored under various culture conditions, including T75 tissue culture flasks, nanoporous Transwell inserts, PDMS membranes, and gut‐on‐a‐chip microdevices. Phase‐contrast images were acquired using an inverted microscope (DMi1; Leica Microsystems) equipped with 10× (NA 0.22) and 20× (NA 0.30) objectives, a digital camera (MC120 HD; Leica), and Leica Application Suite software (LAS v4.12; Leica). For each condition and time point, images were captured from over 10 random fields of view across at least two independent biological replicates. Representative images were selected for the figure presentation.

For immunofluorescence staining, cells were fixed with 4% (wt vol^−1^) paraformaldehyde (PFA; Thermo Fisher Scientific) for 30 min, permeabilized with 0.3% (vol vol^−1^) Triton X‐100 (Millipore Sigma) for 15 min, and blocked with 2% (wt vol^−1^) bovine serum albumin (BSA; Millipore Sigma) in phosphate‐buffered saline (PBS; Gibco) for 1 h at room temperature. All steps included PBS washes between treatments. Primary antibodies targeting α‐smooth muscle actin (α‐SMA; mouse, Abcam, cat. no. ab7817), collagen I (COL I; rabbit, Abcam, cat. no. ab90395), vimentin (mouse, Thermo Fisher, cat. no. MA5‐11883), p‐FAK (rabbit, Invitrogen, cat. no. 700255), and vinculin (mouse, Millipore Sigma, cat. no. V9131) were applied for 1 h at room temperature. Samples were then incubated with Alexa Fluor‐conjugated secondary antibodies (Alexa Fluor 555 goat anti‐rabbit, Abcam, cat. no. ab150078; Alexa Fluor 488 donkey anti‐mouse, Abcam, cat. no. ab150105) for 1 h in the dark. Nuclei and F‐actin were counterstained using 4′,6‐diamidino‐2‐phenylindole dihydrochloride (DAPI, 1 µg mL^−1^; Thermo Fisher, cat. no. 62248) and CruzFluor 647‐conjugated phalloidin (1:500; Santa Cruz Biotechnology, cat. no. sc‐363797). Samples were mounted using Fluoromount medium (Millipore Sigma, cat. no. F6182).

Confocal imaging was performed using a Leica TCS SP8 microscope (Leica Microsystems) equipped with a 25× water immersion objective (NA 0.95), excitation lasers (405 nm, 488 nm, 561 nm, and 633 nm), and a hybrid detector (HyD). Single‐plane and Z‐stack images were acquired and analyzed using Leica LAS X software. Orthogonal projections from Z‐stack images were generated for high‐resolution vertical cross‐sectional analysis. Fluorescence intensities were quantified from three randomly selected areas per condition, and cell layer thickness was measured from 10 cross‐sectional views using ImageJ (NIH).

For longitudinal live‐cell monitoring (Figure 4A,B), nFib cells were labeled using CellTracker dye (Invitrogen; cat. no. 7025) according to the manufacturer's instructions. Briefly, cells were harvested by trypsinization, resuspended in CellTracker working solution (final concentration, 10 µm), and incubated at 37°C for 30 min. Labeled cells were then washed twice with PBS, resuspended in fibroblast culture medium at a concentration of approximately 1 × 10^7^ cells mL^−1^, and introduced into the upper microchannel of a gut‐on‐a‐chip device. Following cell attachment under static conditions, fluorescence imaging was performed intermittently using an EVOS fluorescence microscope (EVOS M5000; Thermo Fisher Scientific) equipped with a 10× (NA 0.3) objective and an integrated live‐cell monitoring system. To monitor cyclic stretch applied to either a cell‐free or a cell‐laden PDMS membrane in a gut‐on‐a‐chip (Movies S1 and S2), the device setup was temporarily placed on the stage of a phase‐contrast microscope (DMi1; Leica Microsystems) equipped with a 20× (NA 0.30) objective and a digital camera (MC120 HD; Leica), real‐time movies were captured while the device setup was connected to the Flexcell tension system, which applied cyclic deformations. To capture time‐lapse movies monitoring the behavior of nFib cells in response to constant fluid flow (Movies S3 and S4), the gut‐on‐a‐chip setup containing a monolayer of nFib cells was placed in an environmental chamber integrated with a control unit (EVOS Onstage Incubator, OSI‐2; Thermo Fisher Scientific) for temperature and humidity regulation within the EVOS microscope system.

For scanning electron microscopy (SEM) analysis, cells were fixed with 2% (wt vol^−1^) paraformaldehyde (Electron Microscopy Sciences, cat. no. 15710) and 2.5% (wt vol^−1^) glutaraldehyde (Electron Microscopy Sciences, cat. no. 16320) in PBS for 30 min at room temperature, followed by post‐fixation with 1% (wt vol^−1^) osmium tetroxide (Electron Microscopy Sciences, cat. no. 19150) in 0.1 m sodium cacodylate buffer (Electron Microscopy Sciences, cat. no. 11650) for 60 min in a fume hood. Samples were extensively rinsed with PBS, dehydrated through graded ethanol solution (70%, 95%, and 100%, vol vol^−1^; 10 min each), treated with hexamethyldisilazane (HMDS; Electron Microscopy Sciences, cat. no. 16700) for 10 min, and dried overnight in a vacuum desiccator containing anhydrous desiccant (Drierite, 8 mesh; cat. no. 23005). Dried specimens were mounted on aluminum stubs with conductive carbon adhesive tape, sputter‐coated with ∼10 nm layer of gold (Electron Microscopy Sciences), and imaged using a Zeiss SIGMA VP Scanning Electron Microscope (Carl Zeiss Inc.).

The aspect ratio of normal and inflammation‐associated fibroblasts was quantified using fluorescence images visualizing F‐actin and nuclei. For each cell, the longest cellular axis (length, *L*) and the shortest perpendicular axis (width, *W*) were measured using ImageJ software. The aspect ratio (AR) was calculated as the ratio of length to width (AR = *L*/*W*), providing a quantitative measure of cell elongation. For each experimental condition, at least 10 cells were analyzed to obtain a representative average aspect ratio.

Fibroblast alignment in response to fluid shear stress or cyclic strain was assessed by measuring the percentage of cells oriented along the principal strain axis and analyzing their orientational angles relative to this axis. The degree of alignment was determined by calculating the percentage of cells oriented within a defined angular range relative to the direction of applied mechanical force, providing a quantitative measure of fibroblast alignment [67]. Alignment analysis was performed using the Directionality plugin in ImageJ software, which quantifies pixel orientation distributions from fluorescence images and generates histograms representing the predominant cellular alignment.

### 5.6 Mechanobiological Characterization

Mechanical properties of fibroblasts, cell aggregates, and culture substrates were quantified using a high‐performance atomic force microscope (AFM; MFP‐3D‐Bio AFM, Oxford Instruments). Tipless cantilevers (ARROW‐TL1Au, Nanoworld; nominal spring constant, 0.03 N m^−1^) were modified by attaching 5‐µm polystyrene beads (Polysciences, Inc.), as previously described [68]. For live‐cell indentation assays, force‐distance curves were collected from at least 25 randomly selected fibroblasts per condition, using a constant scan rate of 0.15 Hz and a trigger setpoint of 0.3 V. Young's modulus was calculated by fitting force‐indentation data (indentation depth ∼500 nm) to the Hertzian contact model [69]. Nanoindentation assays were similarly conducted on aggregates harvested at day 4 and day 10 of culture. For AFM topography, cells and aggregates were gently fixed with 0.1% (wt vol^−1^) paraformaldehyde for 2 min at room temperature, rinsed with distilled water, and imaged at 0.5 Hz scan rate, setpoint of 0.1 V, and integral gain of 10 over a 90 × 90 µm^2^ scan area.

### 5.7 Cellular Assessment

Oxidative stress and apoptosis were simultaneously evaluated using CellROX Orange Reagent (Thermo Fisher Scientific, cat. no. C10444) and BioTracker NucView 405 Blue Caspase‐3 Dye (MilliporeSigma, cat. no. SCT‐104), respectively. Fibroblasts cultured in a gut‐on‐a‐chip were incubated with a mixture of both dyes (5 µm each) perfused into the apical and basal microchannels at 20 µL h^−1^ in a 5% CO_2_ incubator at 37°C. After 30 min reaction, cells were rinsed with PBS and imaged by live‐cell confocal microscopy. Reactive oxygen species (ROS) and caspase‐3 activity were detected at excitation/emission wavelengths of 555/565 nm and 405/450 nm, respectively. For quantitative analysis, fluorescence intensities were measured from at least three randomly selected fields per condition using ImageJ software. All experiments were performed in technical duplicates and repeated in at least three independent biological replicates.

Fibroblast proliferation was assessed using the Click‐iT EdU Alexa Fluor 488 Imaging Kit (Invitrogen, cat. no. 10337). Fibroblasts cultured under flow alone (Fluidic) or combined flow and mechanical strain (Dynamic) conditions in the gut‐on‐a‐chip were incubated with 50 µm EdU in fibroblast medium for 3 h at 37°C. After labeling, cells were fixed with 3.7% (wt vol^−1^) PFA for 20 min, washed with 3% (wt vol^−1^) BSA in PBS, permeabilized with 0.5% (vol vol^−1^) Triton X‐100 for 30 min, and stained with the Click‐iT reaction cocktail for 30 min in the dark. Nuclei were counterstained with DAPI (1 µg mL^−1^). EdU‐positive cells and total nuclei were imaged by confocal microscopy, and the percentage of proliferative cells was calculated as EdU^+^/total nuclei across at least three randomly selected fields per condition.

### 5.8 Gene Expression Analysis

Total RNA was extracted from fibroblasts using the RNeasy Mini Kit (Qiagen) following the manufacturer's protocol. RNA purity and concentration were assessed with a NanoDrop spectrophotometer (Thermo Fisher Scientific). Complementary DNA (cDNA) was synthesized from isolated RNA using the RT^2^ First Strand Kit (Qiagen; cat. no. 330404). Gene expression profiling in Figure 3 was performed using the TaqMan Array Human Extracellular Matrix & Adhesion Molecules Panel (Thermo Fisher Scientific; cat. no. 4414133). Real‐time PCR was conducted with the TaqMan Gene Expression Master Mix (Applied Biosystems; cat. no. 4369514) on a QuantStudio 3 Real‐Time PCR System (Applied Biosystems). Relative gene expression levels were determined using the 2‐*Δ*CT method, with normalization to HPRT1 as the housekeeping gene [70]. All reactions were performed in technical duplicates and averaged across at least three independent biological replicates.

### 5.9 Protein Quantification

The concentrations of TGF‐β, IL‐11, and COL1A1 were measured using DuoSet ELISA Kits (TGF‐β, R&D Systems, cat. no. DY240‐95; IL‐11, DY218; COL1A1, DY6220‐05) according to the manufacturer's instructions. Standards and samples were added to 96‐well plates pre‐coated with capture antibodies, followed by sequential incubation with biotinylated detection antibodies and streptavidin‐horseradish peroxidase. Signal was developed with a chromogenic substrate (3,3',5,5'‐tetramethylbenzidine, TMB), and absorbance was measured at 450 nm using a microplate reader (Cytation 5 Multi‐Mode Reader; BioTek Instruments). Antigen concentrations were calculated from standard curves. All samples were run in technical duplicates and averaged across at least three independent experiments.

### 5.10 Measurement of Matrix Metalloproteinase Activity

Extracellular pan‐MMP activity was quantified using the MMP Activity Assay Kit (Abcam; cat. no. ab112146) that covers various MMPs including MMP1, 2, 3, 7, 8, 9, 10, 12, 13, and 14 [33]. Cell culture supernatant was collected, centrifuged at 1000× *g* for 10 min, and mixed 1:1 (vol vol^−1^) with 2 mM 4‐aminophenylmercuric acetate (APMA) to activate latent MMPs, followed by incubation at 37°C for 15 min. After addition of the fluorogenic pan‐MMP substrate, fluorescence was measured every 10 min for 90 min at 37°C using a microplate reader (BioTek Instruments) with excitation/emission settings of 490/525 nm. Each condition was assayed in technical duplicates and averaged across at least three independent biological replicates.

### 5.11 Flow Cytometry

Fibroblasts were harvested via trypsinization, washed with 1% (wt vol^−1^) BSA stain buffer (BD Biosciences), blocked with 5 µL Fc block (TruStain FcX, BioLegend; cat. no. 422301), then incubated on ice for 15 min. Cells were then fixed with BD Cytofix fixation buffer (BD Biosciences; cat. no. 554655) for 20 min on ice and permeabilized with BD Perm/Wash Buffer (BD Biosciences; cat. no. 554723). Staining was performed for 30 min at 4°C in the dark using fluorophore‐conjugated antibodies: anti‐CD90‐Brilliant Violet 421 (BioLegend; cat. no. 328114), anti‐vimentin‐Alexa Fluor 488 (BD Biosciences; cat. no. 562338), anti‐α‐SMA‐APC (R&D Systems; cat. no. IC1420A), and anti‐fibronectin‐PE (R&D Systems; cat. no. IC1918P). After staining, cells were washed, resuspended in buffer, and analyzed on a Sony ID7000 Spectral Cell Analyzer (Sony Biotechnology). Data were processed using FlowJo software (Version 10; BD Biosciences). Fluorescence‐minus‐one (FMO) controls and isotype controls were used to establish gating strategies. Marker expression was quantified as the percentage of positive cells.

### 5.12 Computational Simulation

A 2D computational model of laminar flow for an incompressible fluid was developed using COMSOL Multiphysics 5.4. The simulated domain included fluidic channels with heights of 500 µm (upper) and 200 µm (lower), separated by a porous membrane (10 µm pore diameter, 25 µm center‐to‐center spacing) representing the cellular interface. To model low Reynolds number conditions, inertial terms were neglected from the Navier‐Stokes equations. Stationary studies were performed to evaluate flow velocity (µm s^−1^) and shear stress (µPa) distributions under four configurations representing epithelial (30 µm height) and fibroblast (2.5 µm height) layers, based on experimental observations. Cells were modeled as no‐slip wall boundaries. Boundary conditions included fully developed laminar flow at the inlets (100 µL h^−1^ in the top channel; no flow in the bottom channel) and 0 Pa pressure at the outlets with backflow suppression. Velocity fields (“spf.u”, “spf.u.x”, and “spf.u.y”) were visualized using surface and vector plots. Shear stress was calculated by multiplying the shear rate (“spf.sr”) with dynamic viscosity (“spf.mu”), approximated as that of water (8.9 × 10^−4^ Pa·s). Shear stress values were exported as spreadsheets and filtered by x‐ and y‐coordinates to extract data from regions corresponding to the cellular surfaces.

### 5.13 Statistical Analysis

All statistical analyses were performed using GraphPad Prism (version 10; GraphPad Software Inc.). Data in column charts are expressed as the mean ± standard error of the mean (SEM), unless otherwise specified. Data in box plots show median with min‐to‐max whiskers. For comparisons between two groups, either two‐tailed unpaired or paired Student's *t*‐tests or unpaired two‐tailed Welch's *t*‐test were applied, depending on the experimental design. For comparisons involving three or more groups with a single variable, a one‐way analysis of variance (ANOVA) was performed, followed by Tukey's post hoc test for multiple comparisons. For experiments involving two independent variables, a two‐way ANOVA with Sidak's multiple comparisons test was performed. The ECM contraction assays were quantified by calculating the area under the curve (AUC), with statistical differences assessed using a paired *t*‐test. A *p*‐value less than 0.05 was considered statistically significant.

## Author Contributions

S.M. and H.J.K. conceived and designed the study, conducted the experiments, analyzed the data, and wrote and revised the manuscript. N.T. performed and analyzed a computational simulation. Y.C.S. and O.A. analyzed the data. E.G.E. and C.R.K. contributed to the AFM experiment and analysis. All authors reviewed and approved the final manuscript.

## Funding

This work was supported in part by the Kenneth Rainin Foundation Innovator Awards (to H.J.K. & O.A.), the Crohn's and Colitis Foundation of America Senior Research Awards (to H.J.K.), Cleveland Clinic VeloSano Pilot Grants (to H.J.K.), the Bio‐industrial Technology Development Program from the Ministry of Trade, Industry & Energy Korea (20018770; to H.J.K.), the Clinical and Translational Science Collaborative of Northern Ohio funded by the NIH NCATS, Clinical and Translational Science Award (UM1TR004528; to H.J.K.), and the National Science Foundation (1927602, 1337859, and 2042116; to C.R.K.).

## Conflicts of Interest

The authors declare no conflicts of interest.

## Supporting information




**Supporting File 1**: advs75197‐sup‐0001‐SuppMat.docx.

**Supporting File 2**: advs75197‐sup‐0002‐MovieS1.avi.

**Supporting File 3**: advs75197‐sup‐0003‐MovieS2.avi.

**Supporting File 4**: advs75197‐sup‐0004‐MovieS3.mp4.

**Supporting File 5**: advs75197‐sup‐0005‐MovieS4.mp4.

## Data Availability

The data that support the findings of this study are available from the corresponding author upon reasonable request.
