# Supplementary material for: Mechanostimulatory Cues Determine Intestinal Fibroblast Fate and Profibrotic Remodeling in a Physiodynamic Human Gut‐on‐a‐Chip
Source: Adv Sci (Weinh). 2026 Apr 10;13(36):e16040. doi: 10.1002/advs.202516040 (PMC13317784; doi:10.1002/advs.202516040)
Supplement: Supplementary file 1 — Supporting File 1: advs75197‐sup‐0001‐SuppMat.docx. [file ADVS-13-e16040-s004.docx]

Supporting Information

Mechanostimulatory Cues Determine Intestinal Fibroblast Fate and Profibrotic Remodeling in a Physiodynamic Human Gut-on-a-chip

Soyoun Min, Nam Than, Yong Cheol Shin, Elif G. Ertugral, Chandrasekhar R. Kothapalli, Olumuyiwa Awoniyi, and Hyun Jung Kim*

***Correspondence to:**

Hyun Jung Kim, PhD

Department of Inflammation and Immunity, Cleveland Clinic Research

Cleveland Clinic

9500 Euclid Ave.

Cleveland, OH 44195, USA

Phone: +1-216-445-8148

**Email:** [kimh19@ccf.org](mailto:kimh19@ccf.org)


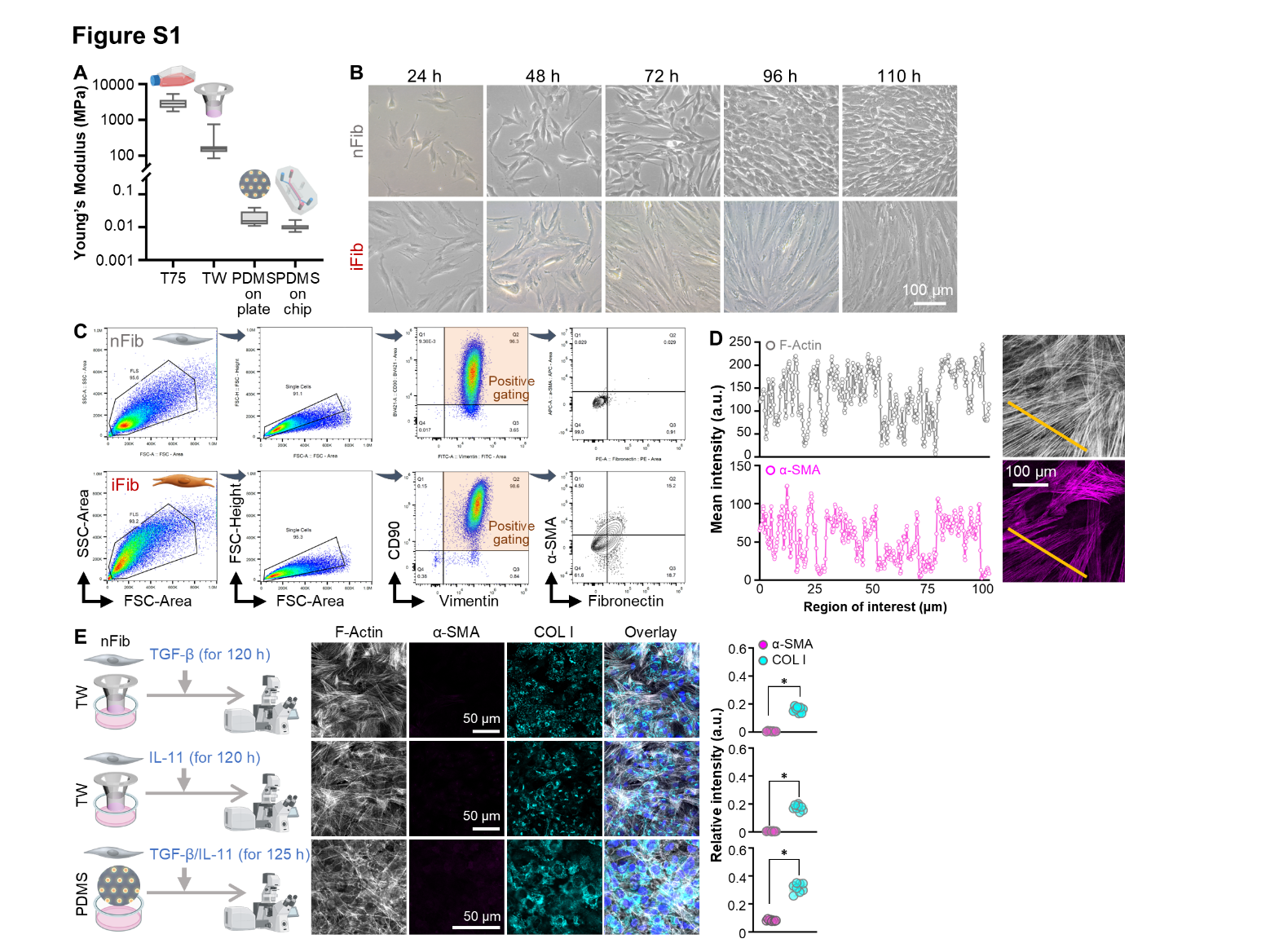


**Figure S1.** Characterization of normal fibroblasts (nFib) and inflammation-associated fibroblasts (iFib) across various culture substrates. A) Young's modulus measurements of different culture substrates, including a T75 flask, a nanoporous polyester membrane in a Transwell (TW), a PDMS membrane (10:1 base polymer:curing agent ratio), and a PDMS membrane fabricated within a Gut-on-a-chip device (*n*=65 per condition). B) Representative phase-contrast images showing morphological profiles of nFib and iFib over time. This duration was selected as the endpoint for baseline characterization based on prior culture optimization. C), Gating strategy for flow cytometry analysis of nFib and iFib cultured in T75 flasks. Cells were first gated based on forward scatter (FSC) and side scatter (SSC) to exclude debris and doublets, followed by gating for CD90 and vimentin expression (orange rectangular gates). Myofibroblast markers, α-SMA and fibronectin, were assessed within the positively gated populations. D) Mean intensity profile from the line scan analysis of immunofluorescence confocal images of iFib cultured on a Transwell insert (related to Figure 1E, lower panel). Orange lines indicate the regions of interest. F-actin (gray); α-SMA (magenta). E) Effect of treatment with profibrotic factors, TGF-β (10 ng mL^-1^) and/or IL-11 (10 ng mL^-1^), on nFib cultured on TW or PDMS membranes under static conditions for 120 h. The schematic (left) depicts the experimental setup. Representative immunofluorescence micrographs show F-actin (gray), α-SMA (magenta), and COL I (cyan) expression, along with overlay images. Quantification of relative fluorescence intensities is shown (right panel) (*n*=4). Data in box plots (S1A) show median with min-to-max whiskers. Data in dot plots (S1E) are presented as mean ± SEM. Statistical significance was determined by one-way ANOVA with Tukey's multiple comparisons test (S1A, S1E). **p*<0.001.


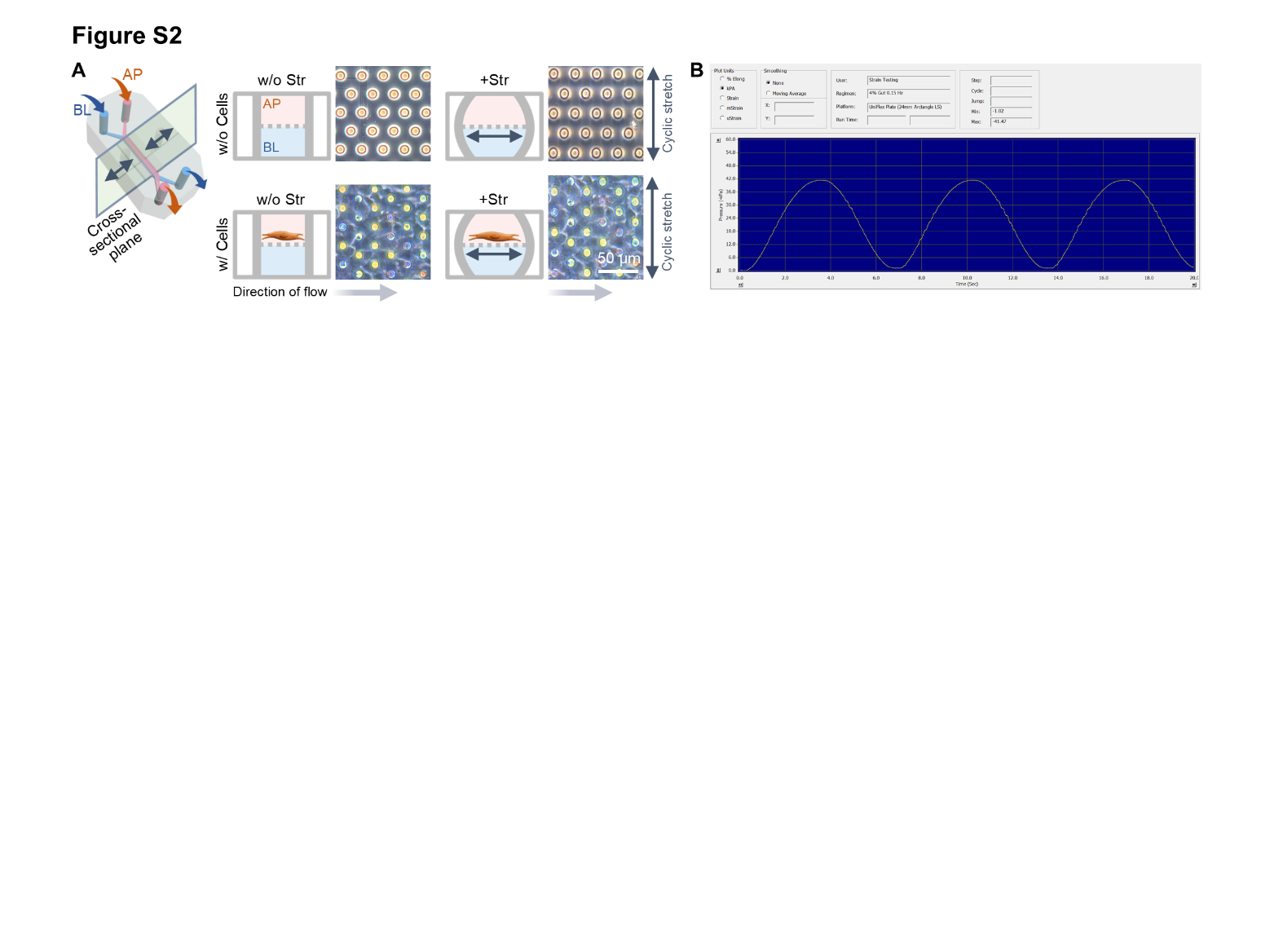


**Figure S2.** Operational mechanism of cyclic stretch in a Gut-on-a-chip microsystem. A) Schematic illustrating the application of biaxial cyclic stretch and unidirectional flow to cells cultured within a Gut-on-a-chip microfluidic device. The top panels show a cross-sectional view of the device without cells under static conditions (w/o Str) and during cyclic stretch (+Str). The bottom panels depict corresponding conditions with cells adherent to the porous membrane substrate. Phase contrast micrographs of cell-free PDMS membranes and membranes with adherent cells are shown. Grey arrows indicate the direction of culture medium flow; blue double-headed arrows represent the direction of cyclic mechanical stretch. B) Sinusoidal waveform depicting the cyclic stretch profile applied to the microfluidic device via a vacuum-driven pneumatic regulator (Flexcell Tension System).


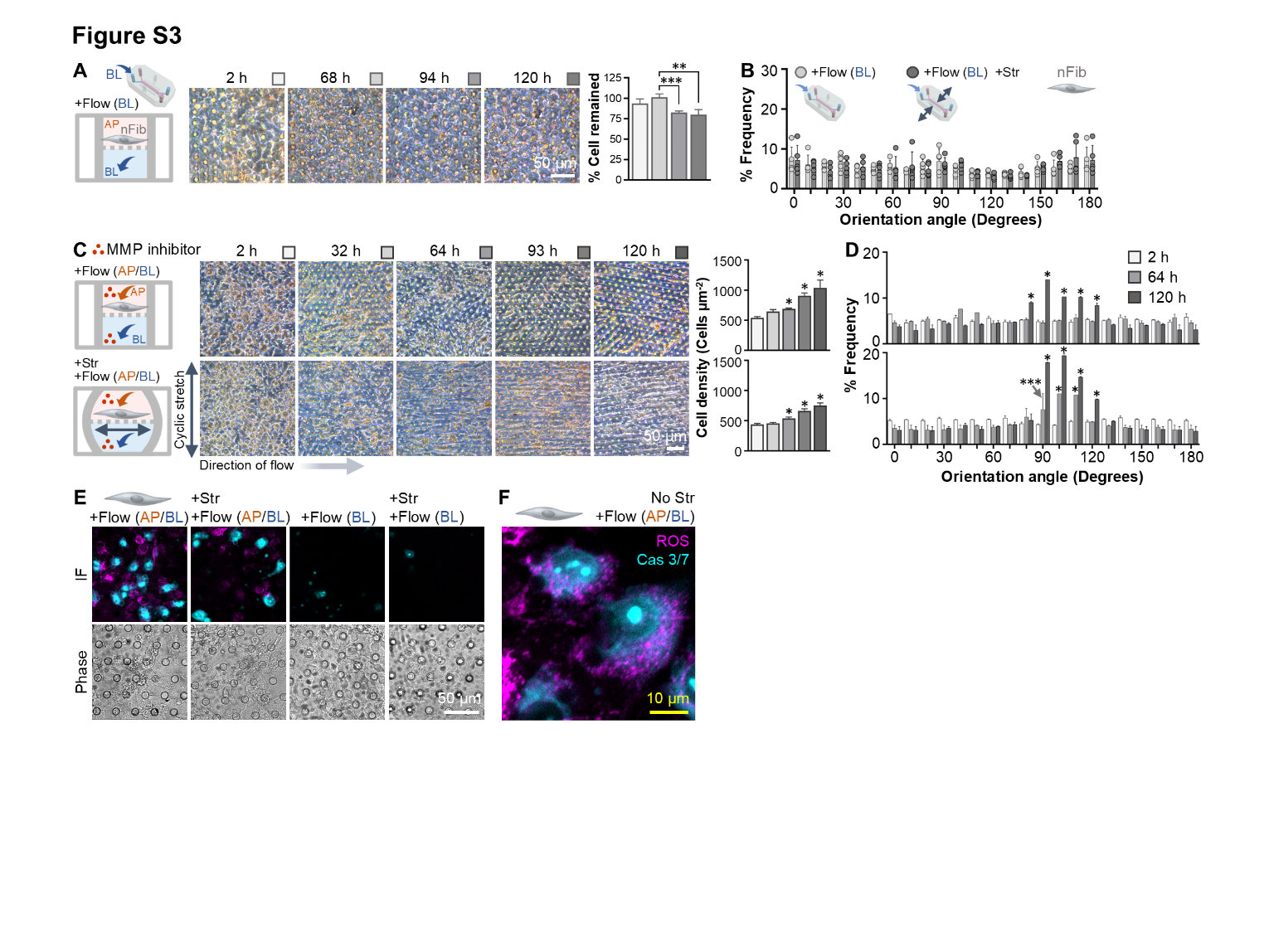


**Figure S3.** Cellular and morphological assessment of nFib under various biomechanical conditions or MMP inhibition. A) Time-lapse phase-contrast images showing nFib morphology over time under pseudostatic basolateral flow (20 µL h^-1^) in the Gut-on-a-chip (+Flow BL). The right panel shows quantification of the percentage of cells remaining at each time point (*n*=3). B) Polar histograms depicting the frequency distribution of orientation angles for nFib cultured in a Gut-on-a-chip under basolateral flow alone (+Flow BL) or combined basolateral flow and cyclic mechanical strain (+Flow BL, +Str), analyzed at 120 h using ImageJ software. C) Representative phase-contrast micrographs of nFib treated with an MMP inhibitor (GM6001, 25 μM) under dual flow conditions (+Flow AP/BL) with or without cyclic strain (+Str). Grey arrows indicate the direction of medium flow. Quantification of cell density over time is shown on the right (*n*=10), with statistical comparisons made relative to cell numbers at 2 h. D) Polar histograms showing the orientation angle distribution of nFib cells cultured in a Gut-on-a-chip treated with an MMP inhibitor (related to C) (*n*=5). E) Representative fluorescence micrographs (upper panels) showing ROS (magenta) and caspase-3/7 (Cas 3/7; cyan) activity, along with corresponding phase-contrast images (lower panels), for nFib cultured under dual flow (+Flow AP/BL) or pseudostatic basolateral flow (+Flow BL) with or without cyclic stretch (+Str) at 120 h. F) High-magnification fluorescence micrographs highlighting ROS and Cas 3/7 signals in nFib challenged with dual flow without cyclic strain at 120 h. Data are presented as mean ± SEM. Statistical significance was by one-way ANOVA with Tukey's multiple comparisons test (S3A), Dunnett's multiple comparisons test, comparing each time point to the 2 h baseline (S3C), or two-way ANOVA with Sidak's multiple comparisons test (S3D). AP, apical; BL, basolateral. **p*<0.001, ***p*<0.01, ****p*<0.05.


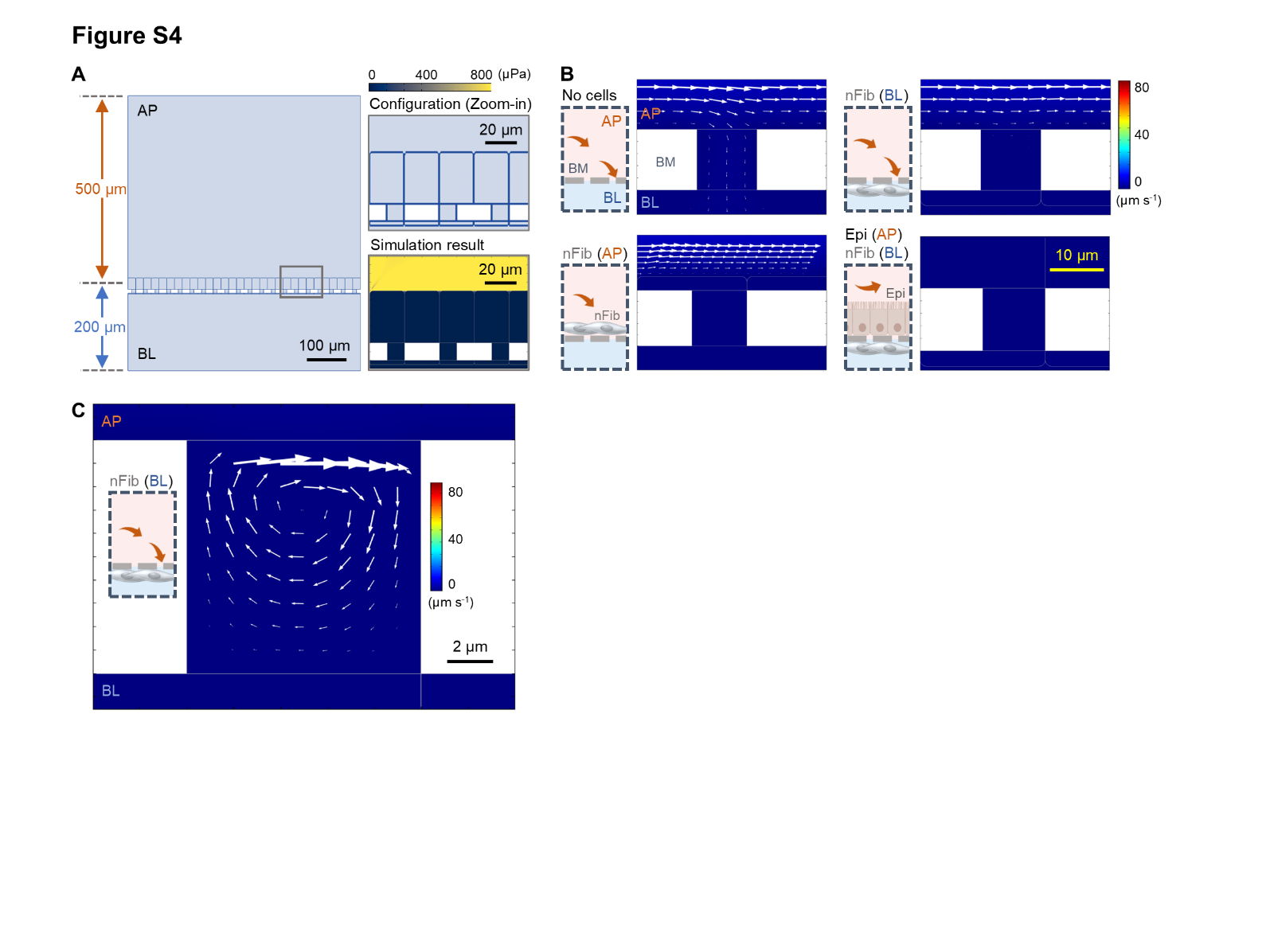


**Figure S4.** Configuration of computational simulations and flow velocity profiles under various culture conditions. A) Schematic illustrations showing a vertical cross-section of the Gut-on-a-chip microchannels and their dimension (left) and a magnified view of the epithelial-nFib interface (top right). A representative simulation result of flow velocity is shown (bottom right). B) Computationally estimated flow velocity profiles for different culture configurations. The size and thickness of white arrows indicate the magnitude of flow velocity. Configurations include: no cells, nFib seeded on the apical side (nFib AP), nFib seeded on the basolateral side (nFib BL), and epithelial cells on the apical side with nFib on the basolateral side (Epi AP, nFib BL). C) Zoomed-in view of the two-dimensional velocity profile in the condition where nFib cells were cultured underneath the porous membrane. Rotational eddies formed under steady-state flow are visualized by local variations in flow velocity. AP, apical; BL, basolateral.


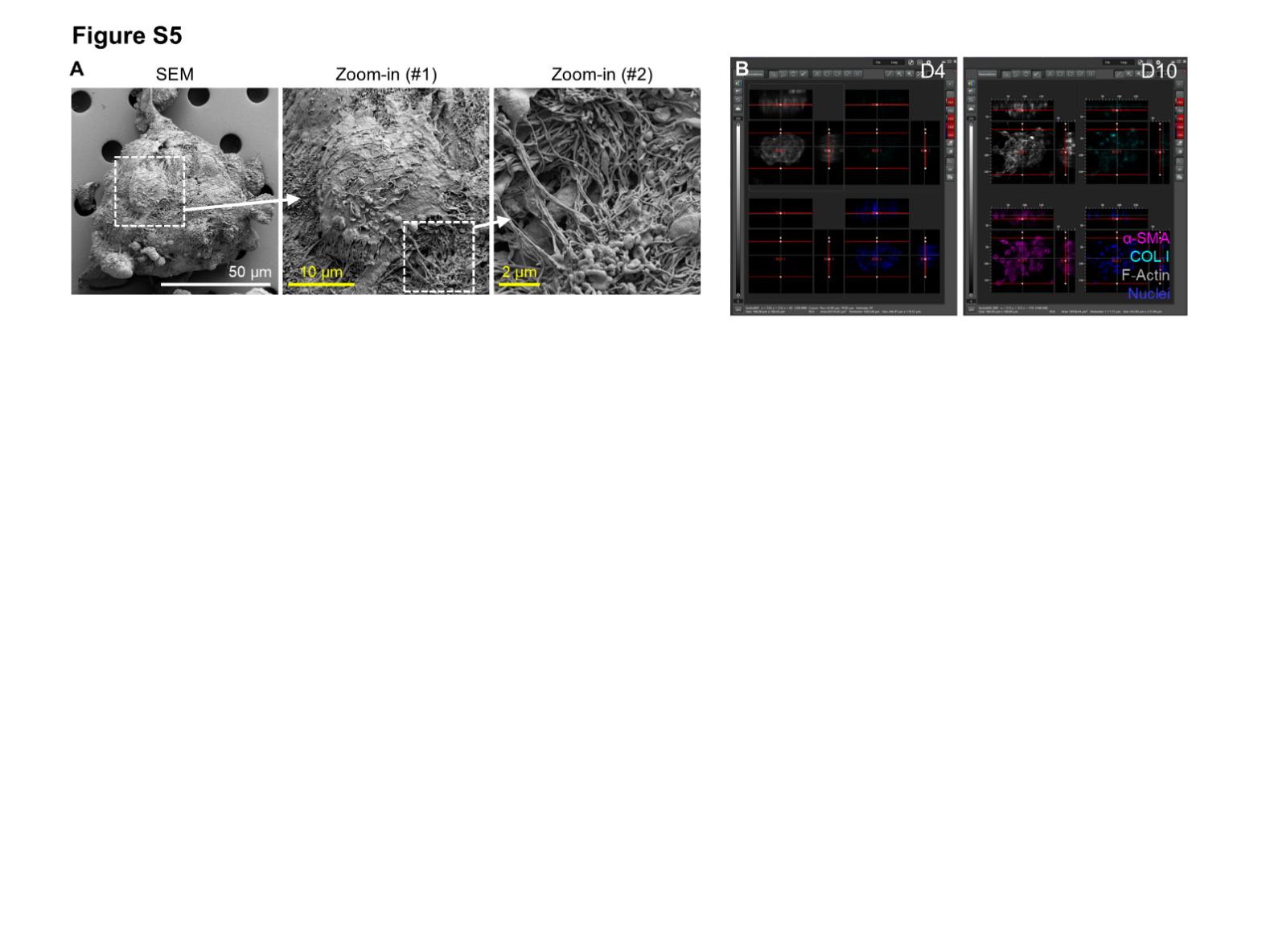


**Figure S5.** Morphological characterization of D10 nFib aggregates. A) Scanning electron microscopy (SEM) images of a D10 nFib aggregate showing the surface morphology and extracellular matrix (ECM) structure. Dashed white squares indicate the region of serial zoom-in views (#1 and #2) that highlight the complex fibrillar network. B) Configurational regions of interest (ROIs) for confocal micrographs of D4 and D10 aggregates (related to Figure 5D and 5E), illustrating the spatial distribution of fibrotic markers, including α-SMA and COL I, counterstained with F-actin and nuclei.


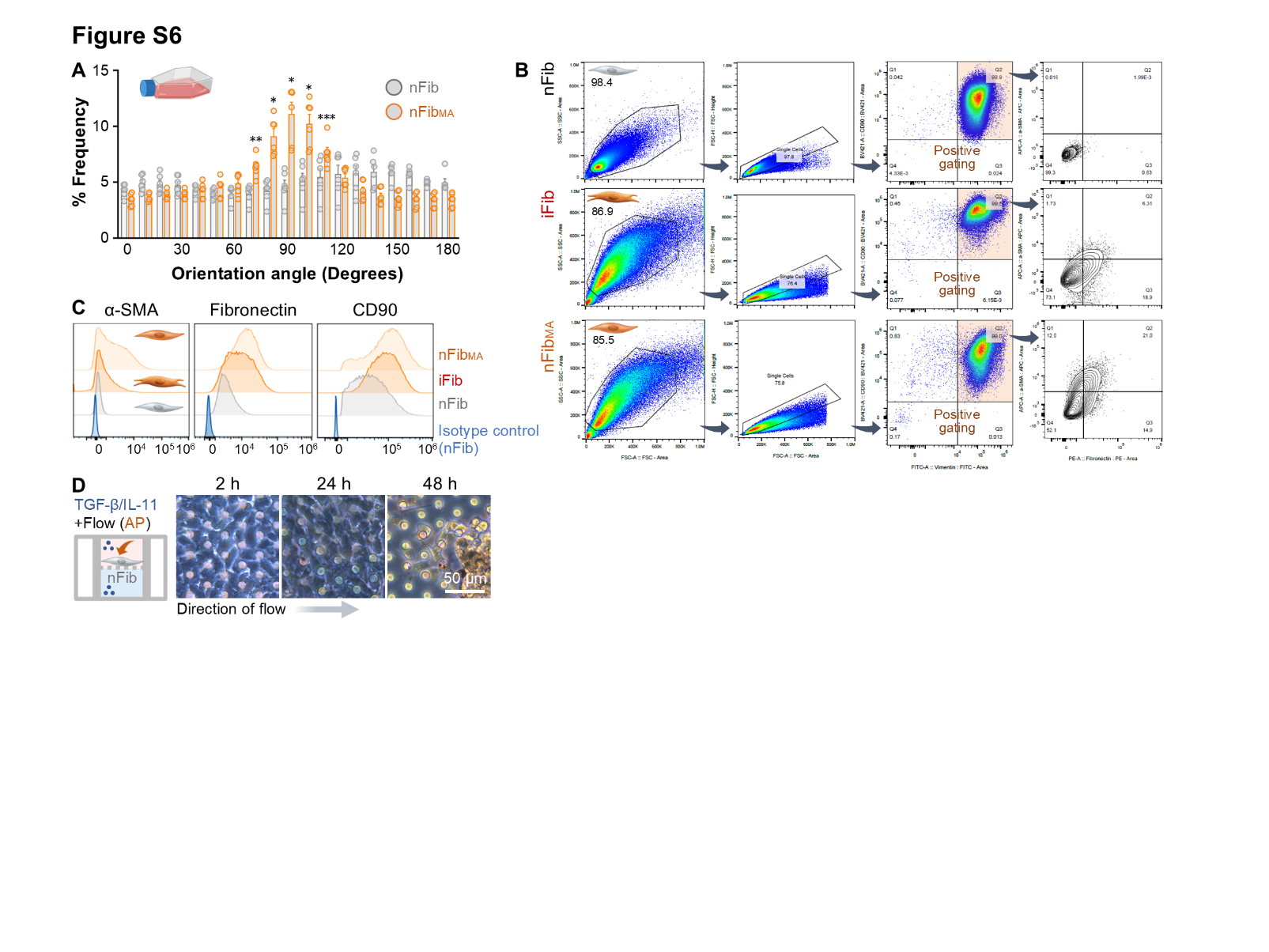


**Figure S6.** Characterization of isolated mechanoadaptive fibroblasts (nFib_MA_). A) Polar histograms illustrating the orientation angle distribution of nFib and nFib_MA_ cultured in T75 flasks for 96 h, analyzed using ImageJ software (*n*=6). B) Gating strategy used for flow cytometry analysis of nFib, iFib, and nFib_MA_ cultured in T75 flasks (96 h). Cells were initially gated by FSC and SSC to exclude debris and doublets, followed by gating for CD90 and vimentin expression (orange rectangular gates). Expression of myofibroblast markers α-SMA and fibronectin was assessed within these positively gated populations. C) Flow cytometry histograms comparing expression profiles of fibrosis-associated markers (α-SMA, fibronectin, and CD90) among nFib, iFib, and nFib_MA_ cultured in T75 flasks. D) Time-lapse phase-contrast micrographs depicting nFib morphology over time under combined IL-11 and TGF-β stimulation (10 ng mL^-1^ each) during apical fluid shear exposure (20 µL h^-1^) in the Gut-on-a-chip for up to 48 h. Data are presented as mean ± SEM. Statistical significance was determined using an unpaired, two-tailed Student's *t*-test (S6A). AP, apical; BL, basolateral. **p*<0.001, ***p*<0.01, ****p*<0.05.

**Legend for Movies**

**Movie S1.** Mechanical deformations of a cell-free PDMS membrane in a Gut-on-a-chip device were subjected to cyclic stretching at 10% strain and a frequency of 0.15 Hz. The repetitive deformation was controlled using a computer-aided tension system.

**Movie S2.** Mechanical deformations of a PDMS membrane, lined with intestinal fibroblast cells in a gut-on-a-chip device, were subjected to cyclic stretching at 10% strain and a frequency of 0.15 Hz. The repetitive deformation was controlled by a computer-controlled tension system.

**Movie S3.** The time-lapse movie demonstrates stochastic shrinkage of nFib cells when exposed to apical shear stress (volumetric flow rate of 20 µL h^-1^, corresponding shear stress of ~0.00133 dyne cm^-2^) in a Gut-on-a-chip for approximately 30 h. Images were captured every 2 min, with the time legend embedded in the video with an hour:minute format (hh:mm). Cells were incubated at 37 ^o^C with 5% CO_2_ in the EVOS incubator system throughout the experiment.

**Movie S4.** The time-lapse movie shows the aggregation of nFib cells when exposed to continuous apical shear stress (volumetric flow rate of 20 µL h^-1^, corresponding shear stress of ~0.00133 dyne cm^-2^) in a Gut-on-a-chip for approximately 50 h. Images were captured every 2 min, with the time legend embedded in the video with an hour:minute format (hh:mm). Cells were incubated at 37 ^o^C with 5% CO_2_ in the EVOS incubator system throughout the experiment.
